# SOCS3 is Related to Cell Proliferation in Neuronal Tissue: An Integrated Analysis of Bioinformatics and Experiments

**DOI:** 10.3389/fgene.2021.743786

**Published:** 2021-09-27

**Authors:** Yeuni Yu, Soon Ki Sung, Chi Hyung Lee, Mihyang Ha, Junho Kang, Eun Jung Kwon, Ji Wan Kang, Youngjoo Kim, Ga Hyun Kim, Hye Jin Heo, Hansong Lee, Tae Woo Kim, Yoonsung Lee, Kyungjae Myung, Chang-Kyu Oh, Yun Hak Kim

**Affiliations:** ^1^ Department of Biomedical Informatics, School of Medicine, Pusan National University, Busan, South Korea; ^2^ Department of Neurosurgery, Pusan National University Yangsan Hospital, Yangsan, South Korea; ^3^ Interdisciplinary Program of Genomic Science, Pusan National University, Yangsan, South Korea; ^4^ Department of Anatomy, School of Medicine, Pusan National University, Yangsan, South Korea; ^5^ Department of Orthopaedic Surgery, Pusan National University Yangsan Hospital, Pusan National University School of Medicine, Yangsan, South Korea; ^6^ Center for Genomic Integrity, Institute for Basic Science (IBS), Ulsan, South Korea; ^7^ Department of Core Research Laboratory, Clinical Research Institute, Kyung Hee University Hospital at Gangdong, Seoul, South Korea; ^8^ Department of Anatomy, College of Medicine, Inje University, Busan, South Korea

**Keywords:** GBM, SOCS3, proliferation, zebrafish, development

## Abstract

Glioma is the most common primary malignant tumor that occurs in the central nervous system. Gliomas are subdivided according to a combination of microscopic morphological, molecular, and genetic factors. Glioblastoma (GBM) is the most aggressive malignant tumor; however, efficient therapies or specific target molecules for GBM have not been developed. We accessed RNA-seq and clinical data from The Cancer Genome Atlas, the Chinese Glioma Genome Atlas, and the GSE16011 dataset, and identified differentially expressed genes (DEGs) that were common to both GBM and lower-grade glioma (LGG) in three independent cohorts. The biological functions of common DEGs were examined using NetworkAnalyst. To evaluate the prognostic performance of common DEGs, we performed Kaplan-Meier and Cox regression analyses. We investigated the function of SOCS3 in the central nervous system using three GBM cell lines as well as zebrafish embryos. There were 168 upregulated genes and 50 downregulated genes that were commom to both GBM and LGG. Through survival analyses, we found that SOCS3 was the only prognostic gene in all cohorts. Inhibition of SOCS3 using siRNA decreased the proliferation of GBM cell lines. We also found that the zebrafish ortholog, socs3b, was associated with brain development through the regulation of cell proliferation in neuronal tissue. While additional mechanistic studies are necessary, our results suggest that SOCS3 is an important biomarker for glioma and that SOCS3 is related to the proliferation of neuronal tissue.

## Introduction

Gliomas are the most common primary malignant central nervous system tumors that originate from glial stem or progenitor cells. Gliomas constitute 31% of all brain tumors, and 81% of all malignant brain tumors are diagnosed in the United States (Ostrom et al., 2015; M et al., 2018). The average annual incidence rate of glioblastoma (GBM) is approximately 5 per 100,000 persons (Ostrom et al., 2016; Strickland and Stoll, 2017). Following the World Health Organization (WHO) classification, gliomas can be subdivided into grades I through IV according to a combination of microscopic morphological, molecular, and genetic factors (Wesseling and Capper, 2018). Traditionally, infiltrating gliomas are classified into grades II–IV depending on the findings of nuclear atypia, proliferative activity, microvascular proliferation, and necrosis (Louis et al., 2007). Glioma could be divided into Low-grade glioma (LGG) and glioblastoma (GBM) based on the histological grade (Louis et al., 2016). LGG is grade II-III glioma and GBM is grade IV glioma. Patients with LGG have a good prognosis and overall survival (OS) is about 5–10 years (Ohgaki and Kleihues, 2005). However, LGG still have a definite recurrence rate and the potential to increase the grade of malignancy, since invasion gliomas are difficult to completely excise by surgery. GBM is the most aggressive malignant tumor (Gao et al., 2019). The current standard treatment for gliomas is maximal safe surgical resection, followed by treatment with radiation and the chemotherapy drug temozolomide (Weller et al., 2017). Even with such a multidisciplinary approach and recent therapeutic advances, GBM has shown poor prognosis and a high recurrence rate, especially in patients with invasive gliomas (Hsu et al., 2019). GBM has the lowest OS, with a 5-years survival rate of less than 5% and a median survival duration of 14 months, even with the best therapy (Stupp et al., 2009; Ostrom et al., 2016).

Tumor RNA sequencing (RNA-seq) data have been used to identify prognostic genes or gene signatures, some of which have been incorporated into clinical guidelines (Ha et al., 2019; Pak et al., 2019). Several studies have been conducted on clinical predictors of survival, and the prognostic clinical factors reported so far include age, extent of resection, Karnofsky Performance Scale score, duration of symptoms, and tumor grade in GBM (Lacroix et al., 2001; McGirt et al., 2009). In 2016, the WHO set the grade to reflect certain gene mutations, such as the co-deletion of chromosomes 1p and 19q, O6-methylguanine-DNA methyltransferase (MGMT) promoter methylation, mutations in the isocitrate dehydrogenase (IDH) IDH1/IDH2 genes, EGFR alterations, and telomerase reverse transcriptase promoter and BRAF V600E mutations (Ducray et al., 2011; Aquilanti et al., 2018).

Although many studies have been conducted on prognostic factors, the prognosis of glioma has not improved; hence, continuous research is needed (Pak et al., 2020). Currently, remarkable advancements in high-throughput sequencing technology, such as microarray and next-generation sequencing, have made it possible to quickly analyze biological markers and understand the mechanisms underlying cancer pathologies (Li et al., 2018). Since GBM studies have focused on gene mutations, there is a need to develop prognostic factors using RNA expression data. Detection of survival-related genes in patients with glioma will play a key role in improving survival outcome prediction and treatment methods by determining the role of oncogenes and tumor suppressor genes.

In this study, we first aimed to investigate common genes by extracting them from low-grade glioma (LGG) and GBM study cohorts. We then analyzed the relationship between their expression profiles and prognosis.

## Materials and Methods

### Data of Glioma Patients in the Study

Using the R package TCGAbiolinks (Colaprico et al., 2016), RNA-seq data and clinical data of gliomas were downloaded from The Cancer Genome Atlas (TCGA) database (https://www.cancer.gov/tcga) (McLendon et al., 2008; Brat et al., 2015). Glioma RNA-seq data from the Chinese Glioma Genome Atlas (CGGA) were also included in our analysis (http://www.cgga.org.cn/) (Zhao et al., 2017). We included primary tumor patients and excluded patients that had insufficient clinical information. TCGA GBMs (*n* = 151) and LGGs (*n* = 515) were included in this study. CGGA693 (GBM, *n* = 140; LGG, *n* = 282) and CGGA325 (GBM, *n* = 85; LGG, *n* = 144) from the CGGA were included in this study (Supplementary Table S1).

**FIGURE 1:**
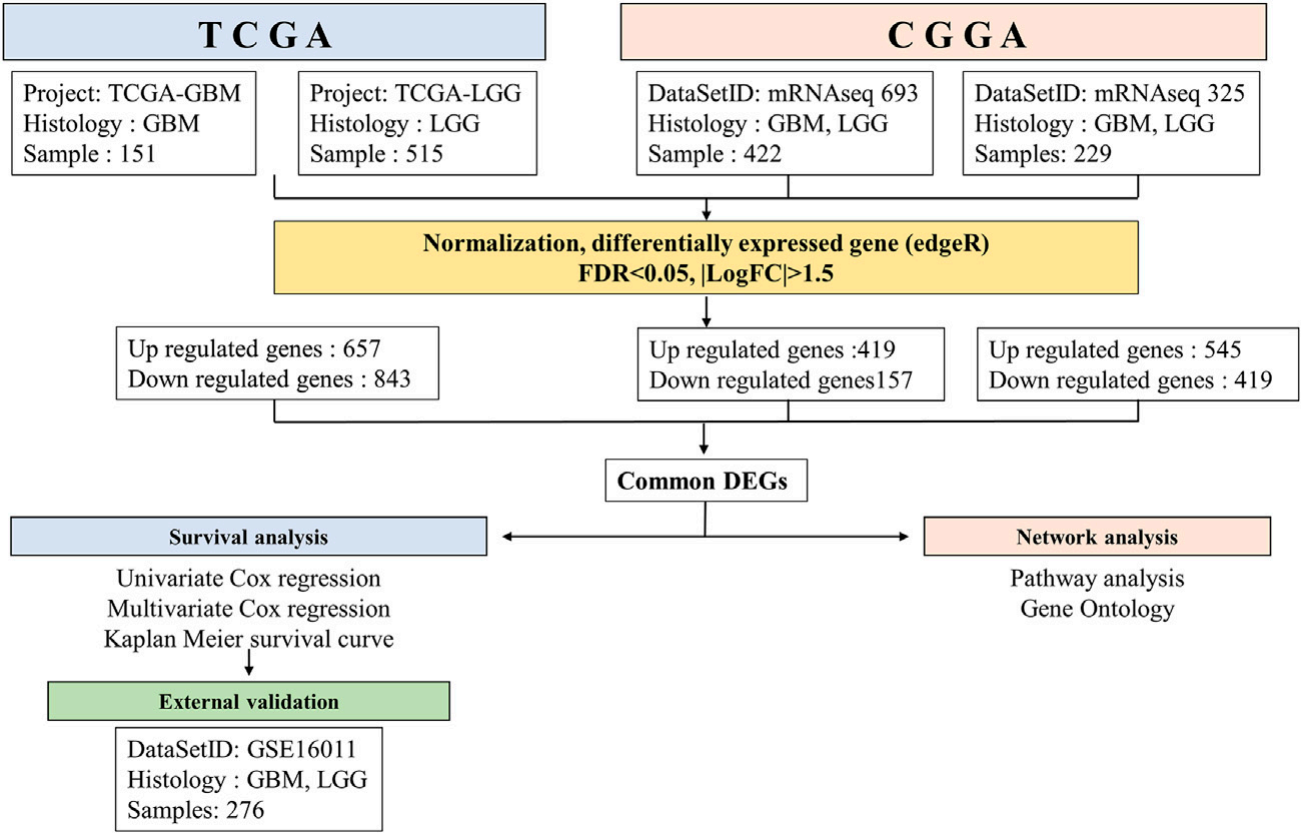
Workflow of gene expression analysis of RNA-seq datasets TCGA and CGGA. Each datasets were run through the pipeline individually, then selected common DEGs between GBM and LGG.

For external validation, we used the GSE16011 dataset (Gravendeel et al., 2019) contains microarray results of brain tissue samples (GBM, *n* = 159; LGG, *n* = 117) in the Gene Expression Omnibus (GEO). The R package “GEOquery” (Davis and Meltzer, 2007) was used to download the data. The overall process for performing this study is presented in Figure 1.

### Identification of Common Differentially Expressed Genes Between Glioblastoma and Lower-Grade Glioma

To identify significantly common differentially expressed genes (DEGs) between GBM and LGG in three independent cohorts were identified using the “edgeR” R package (Robinson et al., 2010). We used the exact test for difference between LGG and GBM of negative binomial counts. Multiple testing correction was executed using the Benjamini-Hochberg method to obtain the adjusted *p*-value. Genes with |log2 (fold change)| > 1.5, and false discovery rate (FDR) < 0.05, were considered statistically significant DEGs.

### Functional Annotation of Differentially Expressed Genes

To interpret the biological functions of common DEGs, Kyoto Encyclopedia of Genes and Genomes pathway and Gene Ontology enrichment analyses were performed using the online software NetworkAnalyst (https://www.networkanalyst.ca/) (Zhou et al., 2019). FDR <0.05 was set as the cut-off criterion for the analysis.

### Generation of the Correlation Coefficient Table

Spearman’s correlation coefficient (r) values between the expressions of SOCS3 and each of the other genes were determined, and genes with *r* > 0.5 were considered to be statistically correlated with SOCS3.

### Statistical Analysis

To identify the prognostic significance of the expression values of common DEGs as a categorical variable, the patients were divided into two groups according to gene expression. Expression levels higher than the median were classified into the high expression group; otherwise, they were classified into the low expression group. (Jin et al., 2019; Nguyen and Le, 2020; Pak et al., 2020; Wei et al., 2020). We performed univariate Cox regression analysis and multivariate Cox regression analysis in each cohort using the “survival” R package and “geneSA” package. Statistical significance was set at *p* < 0.05. A Kaplan-Meier survival curve was constructed based on the median gene expression of each GBM and LGG cohort. The log-rank test confirmed the statistical significance of survival curves. All statistical analyses were performed using R (v.4.0.3).

### Cell Culture

The human glioblastoma cell lines A172, U-87MG, and U-373MG were purchased from the Korean Cell Line Bank. The cells were cultured in DMEM supplemented with 2 mM l-glutamine and 10% fetal bovine serum (FBS). The cells were incubated in a humidified incubator with 5% CO_2_ at 37°C.

### Small-Interfering RNA Transfection

For SOCS3 knockdown, small-interfering RNA for SOCS3 was purchased from Bioneer Corporation (South Korea) and exhibited specific knockdown efficiencies >50%. Cells were transfected with a non-targeting siRNA (5′- UUC UUC GAA CGU GUC ACG U -3′) for 72 h with Lipofectamine RNAimax, according to the manufacturer’s protocol.

### Quantitative Real-Time Polymerase Chain Reaction

Total RNA was extracted using the RNeasy Mini Kit (Qiagen, MD, United States). Complementary DNA (cDNA) was synthesized using a Smart Gene Compact cDNA Synthesis kit (Smart Gene, South Korea). The SOCS3 primers were 5′- CAC CTG GAC TCC TAT GAG AAA GTC A-3’ (forward) and 5′- GGG GCA TCG TAC TGG TCC AGG AA-3’ (reverse), and the GAPDH primers were 5′- CAT GTT CGT CAT GGG GTG AAC CA -3’ (forward) and 5′- AGT GAT GGC ATG GAC TGT GGT CAT -3’ (reverse). Quantitative real-time PCR was performed using the LightCycler 96 Real-Time PCR System (Roche, Risch-Rotkreuz, Switzerland). Expression of target mRNAs relative to housekeeping gene expression (GAPDH) was calculated using the threshold cycle (*C*_T_) as *r* = 2^–Δ(ΔCT)^, where Δ*C*_T_ = *C*_T target_−*C*_T GAPDH_ and Δ(Δ*C*_T_) = Δ*C*_T siSOCS3_–Δ*C*_T Neg_.

### Western Blotting

GBM cells were scraped and homogenized in protein lysis buffer and centrifuged at 13,000 rpm for 10 min at 4 °C. After centrifugation, 30 μg of protein was loaded onto 10% SDS-polyacrylamide gels. Subsequently, the separated proteins were transferred onto nitrocellulose membranes. Membranes were blocked with 5% skim milk and incubated with antibodies against anti-SOCS3 (ABclonal Technology, MA, United States) and PCNA (Santa Cruz, CA, United States) (1:500 dilution). Membranes were probed with an anti-β-actin antibody (ABclonal Technology, MA, United States) as an internal control.

### *In vitro* Proliferation Experiments

Cell viability was determined using MTT [3-(4,5-dimethylthiazol-2-yl)-2,5-diphenyltetrazolium bromide] assays. First, the cells were transfected with siSOCS3 and seeded onto 96-well plates at an initial density of 1 × 10^4^ cells/well. After siSOCS3 transfection for 72 h, 10 μL of MTT (5 mg/ml) was added to each well and the cells were incubated for 4 h. Subsequently, the medium was replaced with 100 μL DMSO to dissolve formazan crystals. The absorbance was measured at 570 nm using a microplate reader (Bio-Rad, Hercules, CA, United States).

### Zebrafish Maintenance and Morpholino Injection

Wild-type AB zebrafish were maintained in an automatic circulation system (Genomic-Design) at 28.5°C. All experiments using zebrafish were performed according to the guidelines of the Ulsan National Institute of Science and Technology (UNIST) Institutional Animal Care and Use Committee (IACUC) (IACUC approval number: UNISTIACUC-15–14, date: 2016–10–11). Zebrafish embryos were cultured in an E3 solution in incubators at 28°C. A translation-blocking morpholino targeting socs3b (Gene Tools, Philomath, OR, United States) was resolved in DEPC water. The sequence of socs3b-MO was 5′-GTC​AAG​CCT​ACT​ATG​CGT​TAC​CAT​G-3'. Morpholinos targeting socs3b were injected into embryos of wild-type AB zebrafish at the 1- or 2-cell stages of development. Microinjections were performed using a FemtoJet 4i microinjector (Eppendorf, Hamburg, Germany).

### *In vitro* Transcription for mRNA Injection

For rescue experiments of socs3b in zebrafish embryos, socs3b mRNA was produced using *in vitro* transcription. After cloning of full-length socs3b in pcs2+ vectors, mRNA was synthesized using mMESSAGE mMACHINE SP6 (Invitrogen, Carlsbad, CA, United States). The synthesized mRNA was injected with a morpholino for rescue experiments.

### Whole-Mount *in situ* Hybridization

WISH was performed in 2 days post-fertilization (dpf) embryos. The overall process for WISH was conducted according to previous studies (Oh et al., 2020; Kang et al., 2020).

### 5-Ethynyl-2′-Deoxyuridine Assay

Zebrafish embryos were saturated with 10 mM EdU for 10 min at 28°C. Embryos were then fixed in 4% paraformaldehyde in PBS. Fixed embryos were dehydrated in methanol at −20°C overnight. After dehydration, embryos were rehydrated in PBS with 0.1% Tween 20 and penetrated with 1% Triton X-100 for 1 h. EdU signals were detected using a Click-iT EdU Alexa Fluor 488 Imaging Kit (Invitrogen, Carlsbad, CA, United States), and samples were imaged using an LSM880 confocal microscope (Carl Zeiss, Oberkochen, Germany).

### Statistical Methods for Zebrafish Experiments

Statistical analysis was performed using Student’s *t*-test, and all experiments were performed in triplicate. The figures and graphs show the averages of three independent experiments. The error bars indicate the standard error of the mean (SEM). A *p*-value less than 0.05 was considered statistically significant.

## Results

### Common Differentially Expressed Genes Between Glioblastoma and Lower-Grade Glioma

**FIGURE 2:**
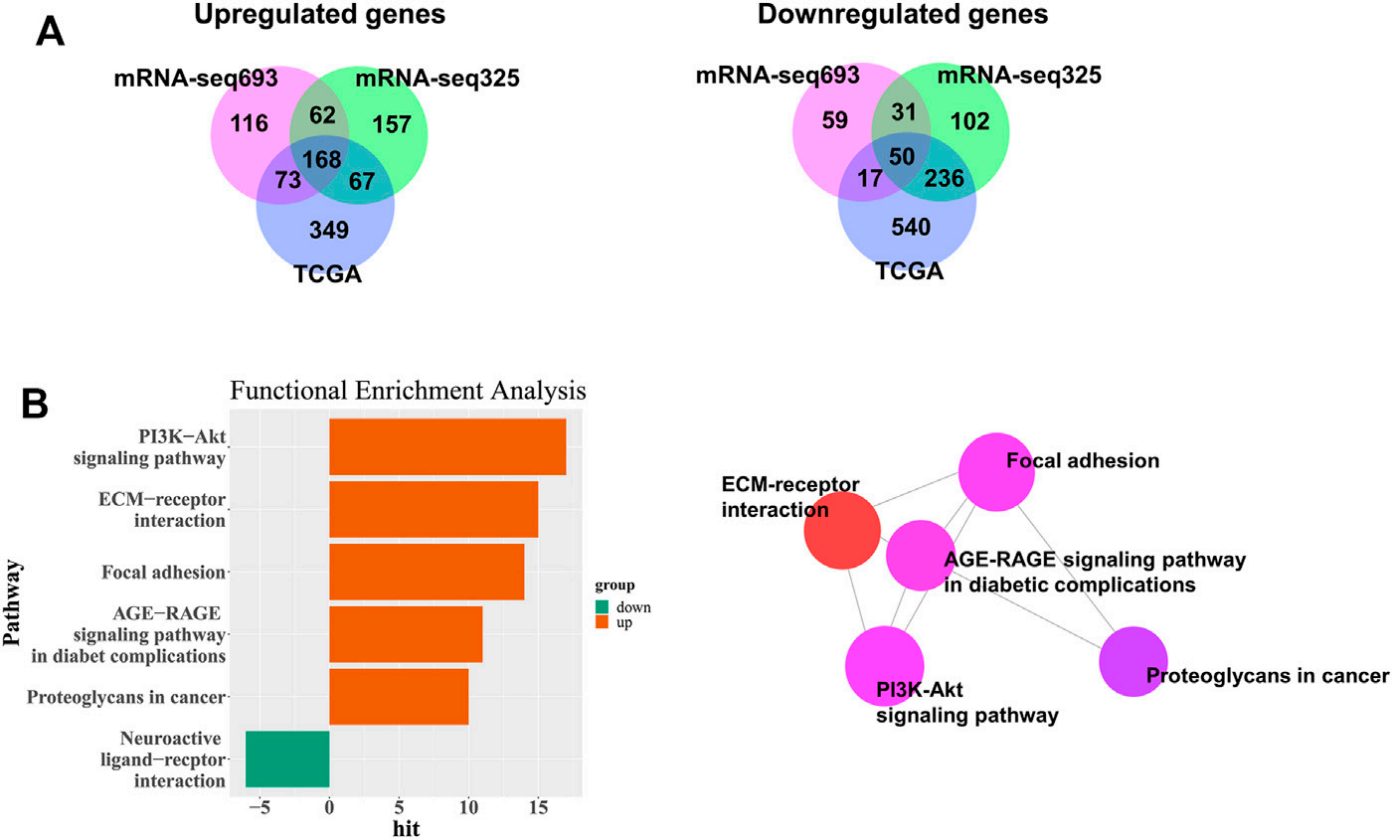
Identification of DEGs among TCGA and CGGA datasets of GBM and LGG. **(A)** Venn diagrams of overlapping DEGs among TCGA, CGGA mRNA-seq325 and CGG mRNA-seq693. **(B)** Significantly enriched biological process and network.

To predict the survival of patients with GBM and LGG, we searched for DEGs in GBM and LGG. A total of 1,500 genes (including 657 upregulated genes and 843 downregulated genes) were identified in the TCGA database. A total of 576 genes (including 419 upregulated genes and 157 downregulated genes) were identified in CGGA693, and 964 genes (including 545 upregulated genes and 419 downregulated genes) in CGGA325 (Figure 2A). Furthermore, 168 upregulated genes and 50 downregulated genes were common to both GBM and LGG (Figure 2A and Supplementary Table S2).

### Functional Enrichment of Common Differentially Expressed Genes

To confirm the potential function of common DEGs, we performed functional enrichment analysis on 168 upregulated and 50 downregulated DEGs. We discovered that the 17 upregulated genes in GBM were functionally associated with the PI3K-AKT signaling pathway (Figure 2B and Supplementary Table S3). The ECM-receptor interaction, focal adhesion, AGE-RAGE signaling pathway in diabetic complications, and proteoglycans in cancer were also associated with upregulated genes in GBM (Figure 2B and Supplementary Table S3), whereas the neuroactive ligand-receptor interaction was associated with GBM downregulated genes (Figure 2B and Supplementary Table S3). The genes involved in each pathway are listed in Supplementary Table S3.

### Suppressor of cytokine signaling 3 is a Prognostic Gene for Glioma and Increased in Glioblastoma

**FIGURE 3:**
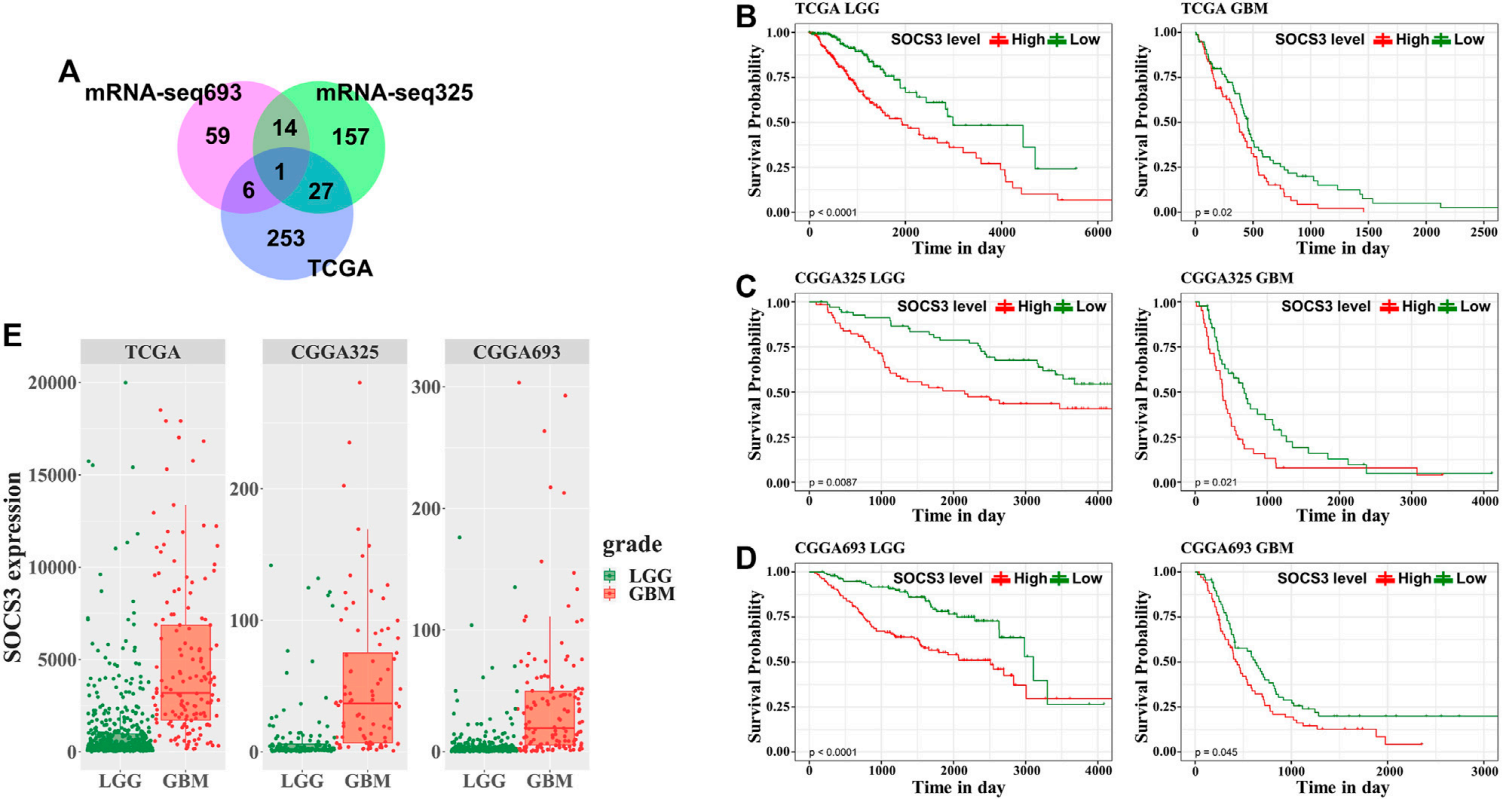
Univariate survival analysis in GBM and LGG stratified by SOCS3 expression based on the TCGA and CGGA data. **(A)** Among common DEGs, SOCS3 was identified as the significant gene by univariate Cox regression. Kaplan-Meier estimates of glioma patient survival according to SOCS3 gene expression from **(B)** TCGA, **(C)** CGGA325, **(D)** and CGGA693. **(E)** Comparison of SOCS3 gene expression between the GBM and LGG cohorts from TCGA, CGGA325 and CGGA693.

To explore the prognostic value of the 218 common DEGs, we used survival analyses to predict OS in patients with GBM and LGG from three independent cohorts. We found that the SOCS3 gene was negatively correlated with OS, and that it was the only statistically significant gene in all cohorts (Figure 3A). Univariate regression analysis in LGG showed that SOCS3 expression, IDH1 wild type, and intact 1p/19q were significantly associated with poor prognosis in all cohorts (Tables 1 and 2). SOCS3 and IDH1 showed similar results in GBM (Tables 1 and 2). Multivariate regression analysis showed that after adjusting for IDH1 mutation and 1p19q co-deletion, SOCS3 expression in LGGs from TCGA still significantly correlated with survival (Tables 1 and 2).

**TABLE 1:** Prognostic value of the SOCS3 gene determined by multivariate Cox regression analysis in LGG.

Cohort	Variable	N	Events	Log-rank test (*p*-value)	HR unadjusted (95% CI)	HR adjusted (95% CI)
TCGA	SOCS3					
	Low	255	35	<0.05	1 (Reference)	1 (Reference)
	High	255	86		2.59 (1.751–3.852)	2.02 (1.15–3.54)
	IDH1					
	Mutant	218	38	<0.05	1 (Reference)	1 (Reference)
	Wild-type	68	30		4.56 (2.79–7.5)	3.9 (2.36–6.52)
	1p19q co-del					
	FALSE	206	57	<0.05	1 (Reference)	1 (Reference)
	TRUE	85	13		0.48 (0.26–0.86)	0.5 (0.3–1.1)
CGGA325	SOCS3					
	Low	71	28	0.015	1 (Reference)	1 (Reference)
	High	69	37		1.857 (1.13–3.1)	1.29 (0.77–2.16)
	IDH1					
	Mutant	103	40	<0.05	1 (Reference)	1 (Reference)
	Wild-type	36	25		3.34 (2.0–5.58)	1.68 (0.97–2.9)
	1p19q co-del					
	FALSE	86	56	<0.05	1 (Reference)	1 (Reference)
	TRUE	52	9		0.15 (0.07–0.31)	0.19 (0.09–0.39)
CGGA693	SOCS3					
	Low	137	32	<0.05	1 (Reference)	1 (Reference)
	High	138	65		2.36 (1.54–3.61)	1.57 (0.96–2.58)
	IDH1					
	Mutant	179	53	<0.05	1 (Reference)	1 (Reference)
	Wild-type	66	38		3.52 (2.29–5.42)	2.0 (1.23–3.3)
	1p19q co-del					
	FALSE	159	73	<0.05	1 (Reference)	1 (Reference)
	TRUE	84	11		0.52 (0.1–0.37)	0.27 (0.13–0.55)
GSE16011	SOCS3					
	Low	58	43	<0.05	1 (Reference)	1 (Reference)
	High	59	49		2.17 (1.41–3.34)	2.32 (1.27–4.23)
	IDH1					
	Mutant	48	41	0.7	1 (Reference)	1 (Reference)
	Wild-type	45	32		1.09 (0.68–1.73)	1.17 (0.66–2.08)
	1p19q co-del					
	FALSE	40	32	<0.05	1 (Reference)	1 (Reference)
	TRUE	38	33		0.49 (0.3–0.82)	0.51 (0.29–0.91)

**TABLE 2:** Prognostic value of the SOCS3 gene determined by multivariate Cox regression analysis in GBM.

Cohort	Variable	N	Events	Log-rank test (*p*-value)	HR unadjusted (95% CI)	HR adjusted (95% CI)
TCGA	SOCS3					
	Low	75	56	0.03	1 (Reference)	1 (Reference)
	High	75	62		1.506 (1.037–2.817)	1.31 (0.9–1.92)
	IDH1					
	Mutant	8	3	0.008	1 (Reference)	1 (Reference)
	Wild-type	139	113		4.667 (1.47–14.77)	4.1 (1.27–13.19)
TCGA325	SOCS3					
	Low	43	34	0.023	1 (Reference)	1 (Reference)
	High	42	39		1.72 (1.08–2.74)	1.58 (0.95–2.62)
	IDH1					
	Mutant	11	9	0.13	1 (Reference)	1 (Reference)
	Wild-type	74	64		1.72 (0.85–3.50)	1.35 (0.63–2.92)
TCGA693	SOCS3					
	Low	68	50	0.03	1 (Reference)	1 (Reference)
	High	68	59		1.54 (1.04–2.21)	1.3 (0.86–1.93)
	IDH1					
	Mutant	23	14	0.02	1 (Reference)	1 (Reference)
	Wild-type	106	91		1.98 (1.12–3.48)	1.79 (0.99–3.22)
GSE16011	SOCS3					
	Low	79	74	0.06	1 (Reference)	1 (Reference)
	High	80	74		1.36 (0.98–1.88)	1.12 (0.77–1.62)
	IDH1					
	Mutant	33	28	0.002	1 (Reference)	1 (Reference)
	Wild-type	95	90		2.04 (1.29–3.22)	2.0 (1.26–3.17)

To evaluate the GBM- or LGG-specific prognostic values of SOCS3, we used Kaplan-Meier curves with median cut-off values (Supplementary Table S4) for SOCS3 gene expression in each cohort (Figures 3B–D). In GBM and LGG, the group with high expression of SOCS3 had a significantly shorter survival than the group with low SOCS3 expression. Additionally, SOCS3 gene expression was higher in GBM than in LGG (Figure 3E, Supplementary Table S4).

### Validation of the Overall Survival

**FIGURE 4:**
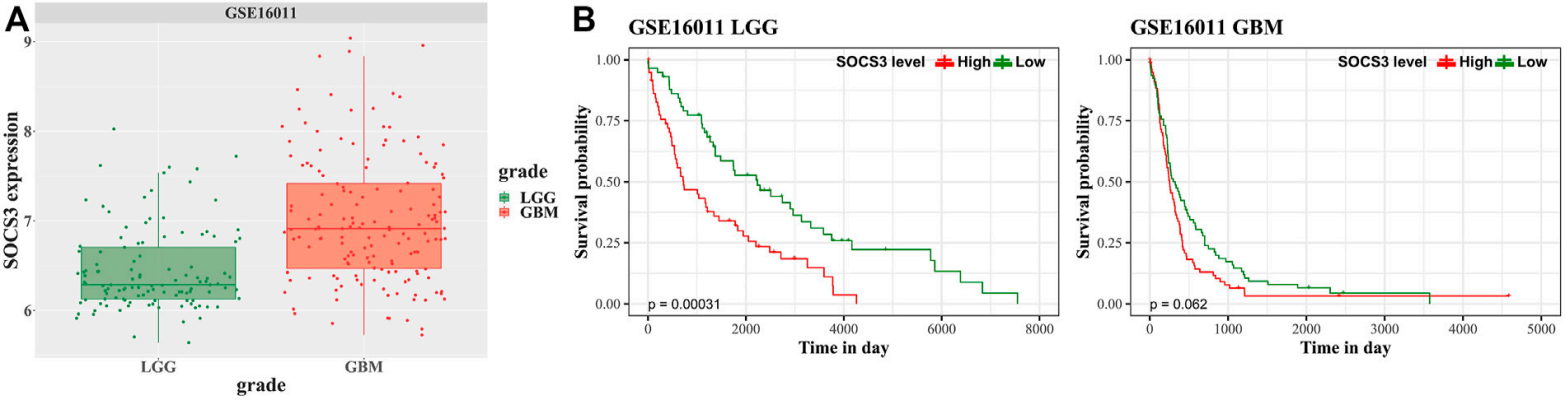
Univariate survival analysis in GBM and LGG stratified by SOCS3 expression based on the GSE16011 dataset for validation. **(A)** Comparison of SOCS3 gene expression between the GBM and LGG cohorts from GSE16011. **(B)** Kaplan-Meier estimates of glioma patient survival according to SOCS3 gene expression.

We analyzed an independently acquired microarray dataset GEO16011, which showed results similar to those of the discovery dataset. SOCS3 gene expression was higher in GBM than in LGG (Figure 4A). The prognosis of LGG and GBM according to the expression of SOCS3 was determined based on the correlation between SOCS3 expression and prognosis in the GSE16011 dataset. SOCS3 showed that it was a good predictor for prognosis of LGG not only in TCGA and CGGA, but also in GSE 16011 (Figure 4B, *p* < 0.05). Although high expression of SOCS3 was associated with poor prognosis, it was not statistically significant in GBM (Figure 4B, *p* = 0.062).

### Inhibition of Suppressor of Cytokine Signaling 3 Decreases Proliferation of Glioblastomas

**FIGURE 5:**
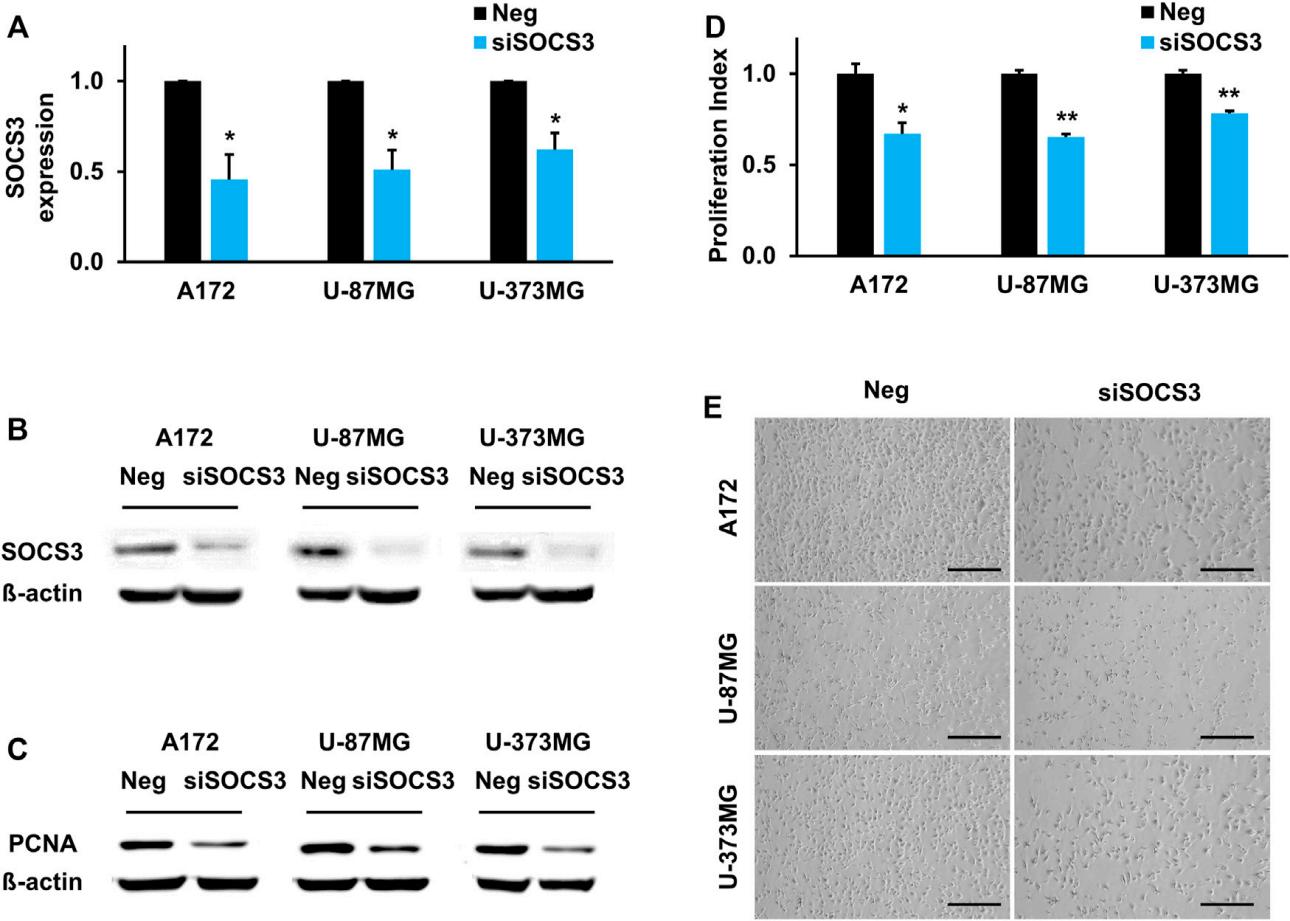
SOCS3 governs brain development by regulating proliferation capacity. **(A,B)** Downregulated SOCS3 expression levels upon transfecting siSOCS3 in human GBM cell lines. **(C)** Comparison of PCNA expression after siSOCS3 transfection by western blot analysis. **(D)** Measurement of cell proliferation rate and corresponding images. Scale bars, 100 µm.

To identify the functional role of SOCS3 in GBM, three GBM cell lines (A172, U-87MG, and U-373MG) were treated with siRNA against the SOCS3 gene. Knockdown of SOCS3 was verified by qPCR analysis and western blotting. Compared to the control siRNA-injected cells, siSOCS3-injected cells showed reduced expression at the transcriptional level (Figure 5A) and translational level (Figure 5B). Since several studies have suggested that SOCS3 is related to cell proliferation (Baus and Pfitzner, 2006; Hackett et al., 2016), PCNA was examined after knockdown of SOCS3 as a cell proliferation marker. Inhibition of SOCS3 expression in three different GBM cell lines showed reduced levels of the PCNA protein (Figure 5C). Additionally, reduced expression of SOCS3 directly reduced cell proliferation (Figures 5D,E). These data suggest that SOCS3 in GBM cell lines is important for cell proliferation.

### Suppressor of Cytokine Signaling 3b is Important for the Proliferation of Neuron Cells in Zebrafish Embryos

In the present study, we showed that SOCS3 is linked to the prognosis of GBM patients using big data analysis. Furthermore, SOCS3 knockdown is related to cell proliferation in GBM cell lines. In previous studies, we showed that several cancer-related genes are involved in vertebrate development (Oh et al., 2020; Kang et al., 2021). In addition to our previous studies, many studies have suggested that signaling pathways in developmental stages and oncology are highly similar. To investigate the function of SOCS3 in the developmental stages of vertebrates, zebrafish embryos were used. The zebrafish embryo is generally used as an *in vivo* model to study neurology or neurodevelopment because of two main advantages (Schmidt et al., 2013; Turner et al., 2014; Venero Galanternik et al., 2017; d'Amora and Giordani, 2018). First, zebrafish embryos are sufficiently transparent to observe the expression of target genes and phenotypes. In addition, since the embryos are transparent, immunofluorescence signals are clearly observed in the target tissue. Second, target genes can be easily manipulated using morpholinos or mRNA injection during the developmental stage. To utilize the zebrafish embryo as an *in vivo* model, a sequence of amino acids was compared between humans and zebrafish. Zebrafish have two kinds of SOCS3 paralogs: socs3a and socs3b. Socs3a showed a 67% similarity of amino acid residues, while socs3b showed a 72% similarity of amino acid residues, indicating that both genes are highly conserved in vertebrates. However, the amino acid sequence was more conserved in socs3b (Supplementary Figures S1A and S1B). Therefore, socs3b was selected as the target gene for knockdown using morpholino injection in zebrafish embryos.

**FIGURE 6:**
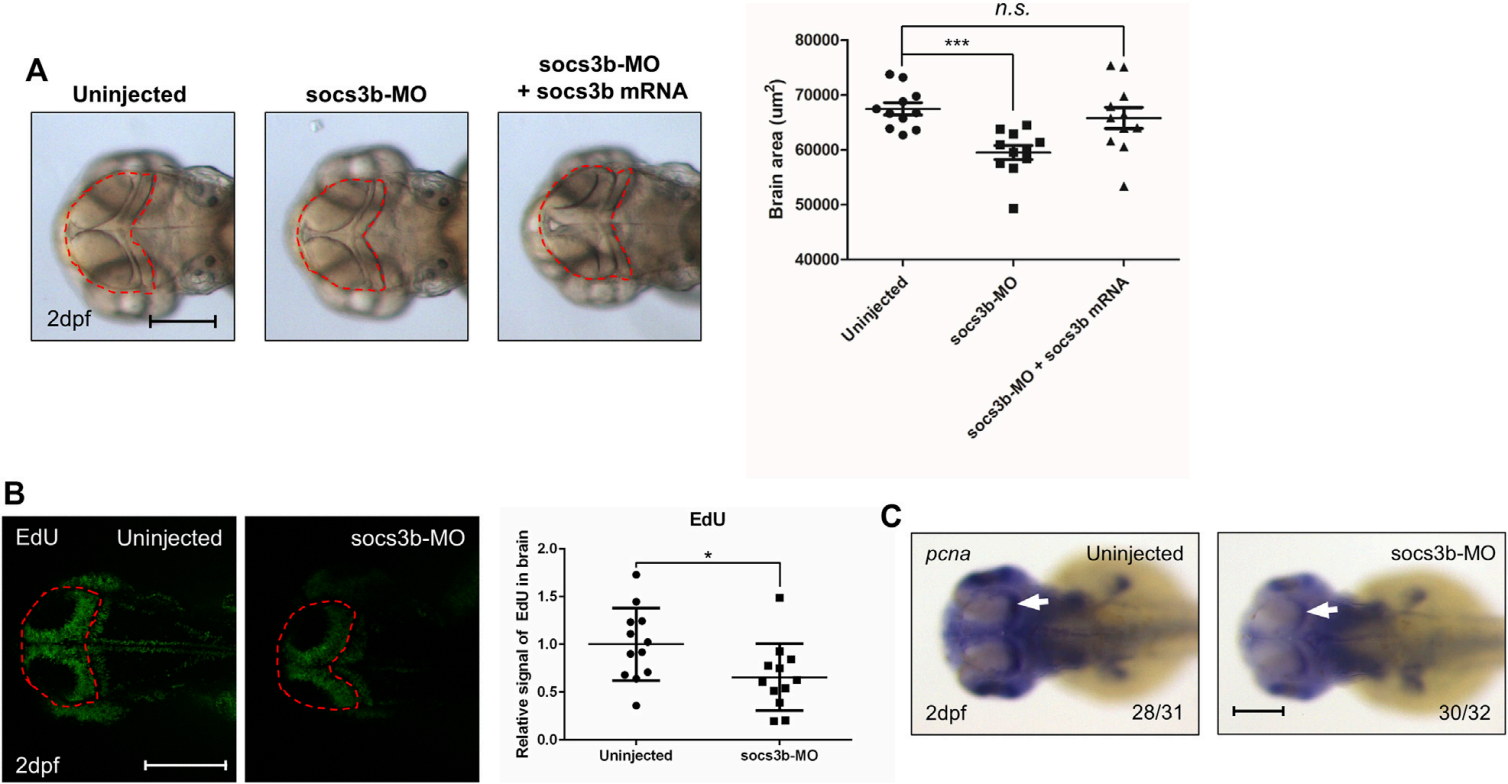
The zebrafish analogue socs3b governs brain development by regulating proliferation. **(A)** Representative images of A172 cells were obtained using a microscope fitted with a digital camera (scale bars, 300 µm). Different concentrations of DHE were added to the culture media of the glioblastoma A172 cells, and cell viability was measured using the MTT assay. **(B)** Dorsal view of live images with uninjected control embryos, socs3b-MO-injected embryos, and socs3b-MO- and socs3b-mRNA-co-injected embryos **(left panel,** red dotted lines indicate brain area), and quantified data of the brain area using ImageJ **(right panel)**. **(C)** Dorsal view of confocal images after EdU staining with uninjected embryos and socs3b-MO-injected embryos **(left panel,** red dotted lines indicate brain area), and quantified intensity of EdU signals **(right panel)**. **(D)** Dorsal view of WISH images using the cell proliferation marker pcna in uninjected embryos and socs3b-MO-injected embryos. Scale bars indicate 200 µm in each image (***p < 0.0001, *p < 0.05).

To explore socs3b function in the brain, socs3b was knocked down using morpholino injection. Because SOCS3 was confirmed in the GBM database, we looked for a brain phenotype. Compared with the control zebrafish embryos, socs3b-MO-injected embryos showed reduced brain size at 2 days post-fertilization (dpf). Brain size was rescued in embryos co-injected with morpholinos and mRNA but not in socs3b-MO-injected embryos (Figure 6A), suggesting that socs3b is linked to neuronal development in zebrafish embryos. As SOCS3 is known to be related to cell proliferation (Xie et al., 2018; Liu et al., 2019), brain proliferation was examined using EdU staining. Compared with control embryos, socs3b-MO-injected embryos showed reduced EdU signals in the brain (Figure 6B). To validate the EdU experiments, the expression level of the cell proliferation marker *pcan* was determined using WISH. Uninjected control embryos showed high levels of pcna in brain areas, especially in the optic tectum, which is known to be a proliferative area (Figure 6C, white arrow). These data suggest that socs3b is important for the proliferation of neurons.

## Discussion

Although several combination treatment methods have been developed to date, glioma remains a malignant brain tumor with a high mortality and recurrence rate. Recent genomic profiling studies have revealed the molecular and signaling pathways involved in the occurrence of GBM, and are therefore expected to improve prognostic factors and treatment methods (Davis, 2016). Due to the growing interest in the important role of molecular biomarkers in tumor prognosis in recent years, we have analyzed survival-related genes associated with GBM prognosis. Based on TCGA, CGGA, and GSE16011 datasets, we integrated and analyzed GBM and LGG samples to identify DEGs in tumor tissues.

Pathway analysis was performed to determine the development of signaling pathways associated with GBM. The results revealed that five pathways may be involved. Several of these identified pathways were consistent with previous reports; for example, the PI3K/AKT signaling pathway was previously reported to induce cell survival and malignant transformation in GBM (Ahn et al., 2004; Koul et al., 2006), while extracellular matrix (ECM) rigidity may mediate the invasion of GBM multiforme cells through actomyosin contractility (Ulrich et al., 2009; Yang et al., 2019). Downregulated DEGs were enriched in neuroactive ligand-receptor interactions. A recent study demonstrated that patients with GBM in combination with a defective neuroactive ligand-receptor interaction pathway have poor prognoses (Pal et al., 2018; Yang et al., 2019).

Among the DEGs that were common to both GBM and LGG, we identified SOCS3 using univariate Cox regression analysis. In the TCGA, CGGA, and GSE16011 datasets, the expression level of SOCS3 was higher in GBM than in LGG, and survival analysis showed that the expression level of SOCS3 had a critical impact on survival time. The prognostic and predictive significance of IDH1/IDH2 mutations has been validated in several studies (Yan et al., 2009; Beiko et al., 2014). In these studies, GBM patients with IDH1/IDH2 mutations had notably longer OS than patients without the mutations. LGG patients with 1p/19q co-deletion had longer survival and better treatment response than patients with 1p/19q intact tumors (Boots-Sprenger et al., 2013; van der Voort et al., 2019). Our results are consistent with those of the previous studies. Although the IDH1/IDH2 mutation and 1p/19q are good prognostic factors in GBM, SOCS3 was significantly correlated with the survival of LGG and GBM patients, regardless of IDH1 status. These results suggest that SOCS3 should be further studied as a prognostic factor in LGG and GBM.

Suppressor of cytokine signaling (SOCS) proteins are intracellular, cytokine-inducible proteins that inhibit the Janus kinase/signal transducer and activator of transcription (JAK/STAT) signal transduction pathway (Mahony et al., 2016). The SOCS family consists of eight proteins: SOCS1 to SOCS7 and cytokine-inducible SH2-containing protein (CIS), each of which contains a central Src-homology 2 (SH2) domain and a specific “SOCS box” at their C-terminal end (Lindemann et al., 2011). Moreover, SOCS1 and SOCS3 contain an additional kinase inhibitory region (KIR) (Tamiya et al., 2011). The KIR and central SH2 domains are major domains in which the SOCS3 protein functions (Dong et al., 2018). In line with these results, SOCS3 is overexpressed in GBM cell lines compared with expression in the normal brain, and plays a critical role in acquiring radioresistance (Zhou et al., 2007). Therefore, SOCS3 may be a new potential prognostic factor that promotes carcinogenesis in gliomas.

Several studies have suggested that SOCS3 acts as a tumor suppressor gene in gliomas. Methylation or silencing of the SOCS3 promoter has been reported to stimulate glioma cell invasion by lowering the expression level of SOCS3 and is also associated with poor clinical outcomes (Martini et al., 2008). However, the role of SOCS3 in suppressing tumors in gliomas remains unclear. Overexpression of SOCS3 has also been reported in GBM cell lines, and hypermethylation of the SOCS3 promoter was associated with better outcomes in GBM patients (Zhou et al., 2007). Feng et al. revealed that hypermethylation of the SOCS3 promoter is only part of the whole genome methylation status, and its negative effect on tumorigenesis or progression may be neutralized by comprehensive genome hypermethylation (Feng et al., 2014).

In this study, we showed poor prognosis in both LGG and GBM when SOCS3 expression was high. Many researchers have identified novel functions of cancer-related genes during development, and we have previously shown that several cancer-related genes are involved in vertebrate development (Oh et al., 2020; Kang et al., 2021). In the present study, we found that SOCS3, a poor prognostic marker of GBM, is important for brain development in vertebrates. Compared to the control zebrafish embryo, SOCS3 knockdown zebrafish displayed reduced brain size, and co-injection with socs3b mRNA rescued the brain size. This result indicated that SOCS3 is related to brain development as well as to the prognosis of GBM. Compared to normal neuronal tissue, growing tumors and early development show a similarity in that cell proliferation is prioritized above all else. In previous studies, we showed that socs3b is associated with the proliferation of neuronal cells at the developmental stage. In addition, inhibition of SOCS3 significantly reduced the proliferation of GBM cells. These data suggest that SOCS3 in GBM is linked to cell proliferation in neuronal tissue. McFarland et al. revealed that loss of SOCS3 in myeloid cells prolongs survival and STAT3 expression in a syngeneic model of glioma. Moreover, this study indicated that loss of SOCS3 decreased tumor formation when compared to control mice (McFarland et al., 2016).

In conclusion, we identified SOCS3 as a predictor for survival in LGG and GBM by analyzing RNA-seq-based gene expression profiles in TCGA, CGGA, and GSE16011 patients. Although the specific mechanism remains to be studied, the Cox proportional hazards regression model and zebrafish experiments demonstrated that the gene could be considered a risk factor for GBM and a target for novel therapeutics.

## Data Availability

The data that support the findings of this study are available at https://www.cancer.gov/tcga and http://www.cgga.org.cn/. The R code in this study is attached in the supplementary file.
