# Supplementary figures and images for: SOCS3 is Related to Cell Proliferation in Neuronal Tissue: An Integrated Analysis of Bioinformatics and Experiments
Source: Front Genet. 2021 Sep 27;12:743786. doi: 10.3389/fgene.2021.743786 (PMC8502821; doi:10.3389/fgene.2021.743786)

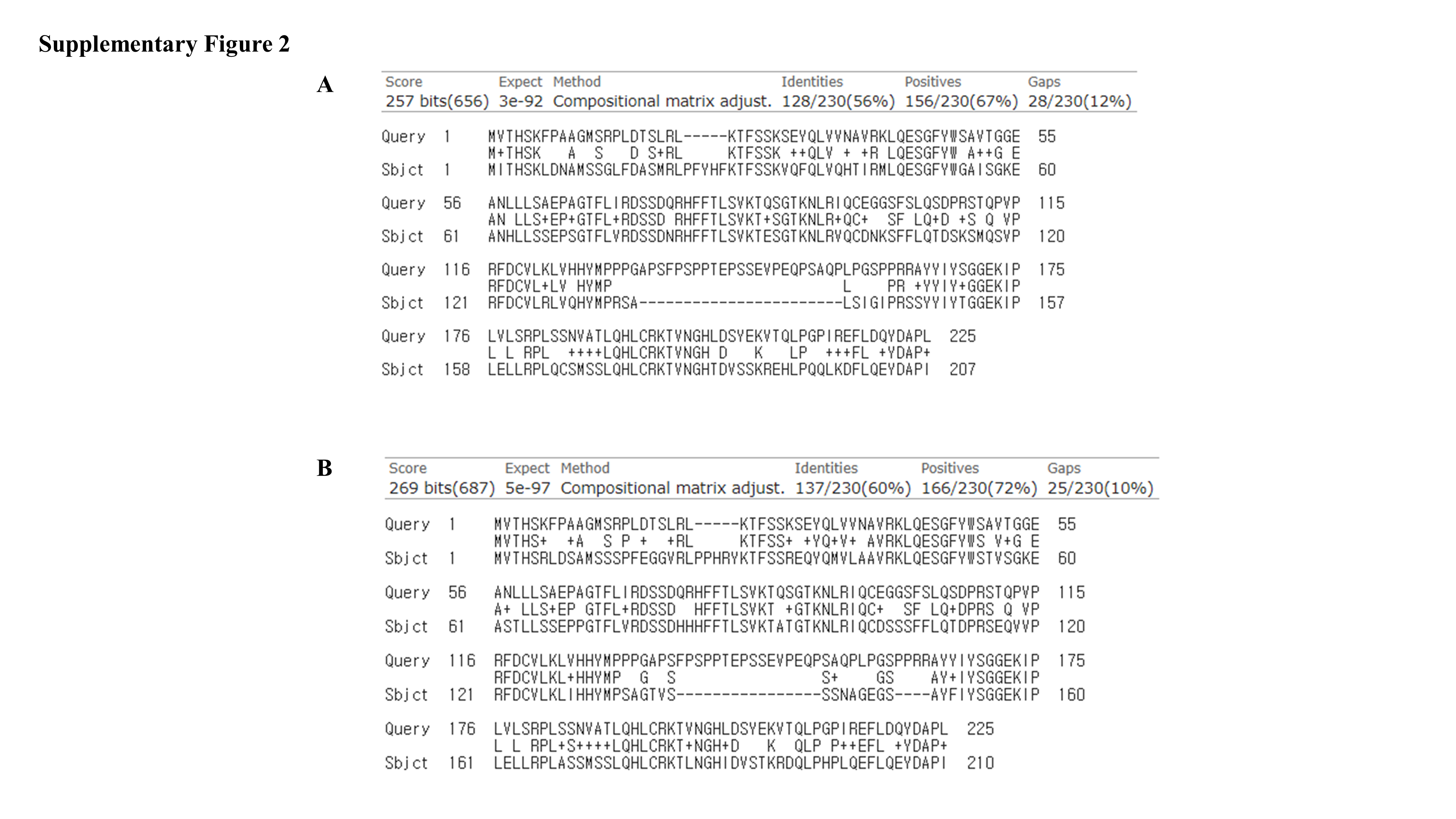

Supplement: Supplementary file 1 [file Image1.TIF]
