# Supplementary material for: SOCS3 is Related to Cell Proliferation in Neuronal Tissue: An Integrated Analysis of Bioinformatics and Experiments
Source: Front Genet. 2021 Sep 27;12:743786. doi: 10.3389/fgene.2021.743786 (PMC8502821; doi:10.3389/fgene.2021.743786)
Supplement: Supplementary file 2 [file DataSheet1.docx]

**Supplementary** **Table 1. Patient characteristics on the included studies**

|  | **TCGA** | **CGGA693** | | **CGGA325** | **GSE16011** |
| --- | --- | --- | --- | --- | --- |
| **No. of patients** | 668 | 422 | | 229 | 276 |
| **Average Age** | 46.8±15.1 | 44±12.8 | | 43.7±12.3 | 50±14.7 |
| **Sex** | | |  | | |
| **Male** | 384(57.5%) | 243(57.6%) | | 142(62.8%) | 184(66.7%) |
| **Female** | 284(42.5%) | 179(42.4%) | | 87(37.2%) | 92(33.3%) |
| **Histology** | | |  | | |
| **GBM** | 153(22.9%) | 140(33.2%) | | 85(37.1%) | 159(57.6%) |
| **LGG** | 515(77.1%) | 282(66.8%) | | 144(62.9%) | 117(42.4%) |
| **Vital status** | | |  | | |
| **Alive** | 418(62.6%) | 205(48.6%) | | 87(38%) | 24(8.7%) |
| **Dead** | 247(37%) | 206(48.8%) | | 138(60.3%) | 240(87%) |
| **Unknown** | 3(0.4%) | 11(2.6%) | | 4(1.7%) | 12(4.3%) |

**Supplementary Table 2. Differentially expressed genes in LGG and GBM in all cohorts**

|  | **Differentially Expressed Genes** |
| --- | --- |
| **Up** | *TUBA1C, LOX, KDELR3, PTX3, VEGFA, CLIC1, PLP2, SPAG4, SERPINH1, LGALS3, TAGLN2, RAB42, COL1A2, OCIAD2, ANPEP, SLPI, PLEK2, IL8, S100A4, BCL3, LUM, TREM1, S100A11, ADM, TDO2, NOX4, PLAUR, GPX8, NNMT, SLC16A3, TIMP1, TNFAIP6, COL6A2, CTHRC1, TGFBI, IGFBP2, ITGA5, LAMC1, PI3, CA9, FBLIM1, COL5A2, COL6A3, SEC61G, ANXA2, IFI30, FCGR2B, HMOX1, MMP14, LOXL1, AQP9, EMP3, S100A9, SDC1, PVT1, ADAM12, SPP1, COL5A1, TFPI2, PHLDA2, STEAP3, LOXL2, FAM20A, TNFRSF12A, PDPN, MYBPH, SERPINE1, CA3, FN1, MMP9, SRPX2, CHI3L2, CDC20, EMILIN1, PLVAP, IGFBP3, SPON2, KCNE4, PRF1, COL4A1, BCAT1, STC1, TAGLN, HK3, S100A8, SERPINA1, HTRA3, PDLIM4, PDLIM1, ANGPT2, METTL7B, COL1A1, CLEC5A, RBP1, ENPEP, GDF15, LAMB1, UBE2C, CHI3L1, ANGPTL4, LIF, PLAU, F13A1, COL4A2, G0S2, IGFBP5, HOXB2, CLCF1, MYBL2, MYO1G, ISG20, C6orf141, CD93, KIF20A, COL3A1, PITX1, THBS1, SOCS3, ANXA1, ABCC3, C1R, VIM, NKX2-5, C5AR1, MMP7, IL2RA, FCGR2C, FOSL1, MXRA5, HSPG2, CHRNA9, ESM1, PLA2G2A, PLA2G5, MEOX2, BCL2A1, RNASE2, IL13RA2, GJB2, THBD, CD163, LTF, IGF2BP2, COL8A1, STAC, ACTG2, SAA1, SPOCD1, CCL18, HP, POSTN, FCGBP, IL1RN, IBSP, HOXD11, SAA2, MARCO, HOXC6, DKK1, ABCA13, EN1, HOXC10, HOXA4, IL6, HOXD13, SLN, GALNT5, H19* |
| **Down** | *CSMD3, SLC22A6, FAM133A, SPHKAP, CDHR1, CALN1, CRTAC1, ATOH8, NDRG2, PRLHR, ZDHHC22, SCG3, FSTL5, HPSE2, RASL10A, TNR, KCNIP2, KCNJ11, CACNG2, HAR1A, MGAT4C, USH1C, DGCR6, HRH3, ABCC8, F5, CSDC2, TRIM67, SMOC1, GDF10, ALDOC, SELL, SYCE1, GABRD, ST8SIA3, WNT7B, RGR, SFRP2, GRIN1, SNCG, SRRM3, CABP1, NTSR2, ACTL6B, SST, MKX, LHX5, IGFN1, CALY, PCDHGB4* |

**Supplementary Table 3. The results of the enrichment analysis using common DEGs**

| **Pathway** | | **Official Symbol** | **Official Full Name** |
| --- | --- | --- | --- |
| **U P** | **PI3K-AKT signaling pathway** | *COL6A3* | Collagen Type VI Alpha 3 Chain |
|  |  | *COL6A2* | Collagen type VI alpha 2 chain |
|  |  | *COL1A1* | Collagen type I alpha 1 chain |
|  |  | *LAMB1* | Laminin subunit beta 1 |
|  |  | *FN1* | Fibronectin 1 |
|  |  | *COL1A2* | Collagen type I alpha 2 chain |
|  |  | *ITGA5* | Integrin subunit alpha 5 |
|  |  | *LAMC1* | Laminin subunit gamma 1 |
|  |  | *COL4A2* | Collagen type IV alpha 2 chain |
|  |  | *THBS1* | Thrombospondin 1 |
|  |  | *COL4A1* | Collagen type IV alpha 1 chain |
|  |  | *SPP1* | Secreted phosphoprotein 1 |
|  |  | *IBSP* | Integrin binding sialoprotein |
|  |  | *VEGFA* | Vascular endothelial growth factor A |
|  |  | *IL6* | Interleukin 6 |
|  |  | *IL2RA* | Interleukin 2 receptor subunit alpha |
|  |  | *ANGPT2* | Angiopoietin 2 |
|  | **ECM-receptor interaction** | *COL6A3* | Collagen Type VI Alpha 3 Chain |
|  |  | *COL6A2* | Collagen type VI alpha 2 chain |
|  |  | *COL1A1* | Collagen type I alpha 1 chain |
|  |  | *LAMB1* | Laminin subunit beta 1 |
|  |  | *FN1* | Fibronectin 1 |
|  |  | *COL1A2* | Collagen type I alpha 2 chain |
|  |  | *ITGA5* | Integrin subunit alpha 5 |
|  |  | *HSPG2* | Heparan sulfate proteoglycan 2 |
|  |  | *LAMC1* | Laminin subunit gamma 1 |
|  |  | *SDC1* | Syndecan 1 |
|  |  | *COL4A2* | Collagen type IV alpha 2 chain |
|  |  | *THBS1* | Thrombospondin 1 |
|  |  | *COL4A1* | Collagen type IV alpha 1 chain |
|  |  | *SPP1* | Secreted phosphoprotein 1 |
|  |  | *IBSP* | Integrin binding sialoprotein |
|  | **Focal adhesion** | *COL6A3* | Collagen Type VI Alpha 3 Chain |
|  |  | *COL6A2* | Collagen type VI alpha 2 chain |
|  |  | *COL1A1* | Collagen type I alpha 1 chain |
|  |  | *LAMB1* | Laminin subunit beta 1 |
|  |  | *FN1* | Fibronectin 1 |
|  |  | *COL1A2* | Collagen type I alpha 2 chain |
|  |  | *ITGA5* | Integrin subunit alpha 5 |
|  |  | *LAMC1* | Laminin subunit gamma 1 |
|  |  | *COL4A2* | Collagen type IV alpha 2 chain |
|  |  | *THBS1* | Collagen type IV alpha 2 chain |
|  |  | *COL4A1* | Collagen type IV alpha 1 chain |
|  |  | *SPP1* | Secreted phosphoprotein 1 |
|  |  | *IBSP* | Integrin binding sialoprotein |
|  |  | *VEGFA* | Vascular endothelial growth factor A |

| **Pathway** | | **Official Symbol** | **Official Full Name** |
| --- | --- | --- | --- |
| **U P** | **AGE-RAGE signaling pathway in diabet complications** | *COL1A1* | Collagen type I alpha 1 chain |
|  |  | *FN1* | Fibronectin 1 |
|  |  | *COL1A2* | Collagen type I alpha 2 chain |
|  |  | *COL4A2* | Collagen type IV alpha 2 chain |
|  |  | *COL4A1* | Collagen type IV alpha 1 chain |
|  |  | *COL3A1* | Collagen type III alpha 1 chain |
|  |  | *SERPINE1* | Plasminogen activator inhibitor-1 |
|  |  | *VEGFA* | Vascular endothelial growth factor A |
|  |  | *NOX4* | NADPH oxidase 4 |
|  |  | *THBD* | Thrombomodulin |
|  |  | *IL6* | Interleukin 6 |
|  | **proteolycans in cancer** | *FN1* | Fibronectin 1 |
|  |  | *ITGA5* | Integrin subunit alpha 5 |
|  |  | *HSPG2* | Heparan sulfate proteoglycan 2 |
|  |  | *SDC1* | Syndecan 1 |
|  |  | *THBS1* | Thrombospondin 1 |
|  |  | *VEGFA* | Vascular endothelial growth factor A |
|  |  | *PLAUR* | Plasminogen activator, urokinase receptor |
|  |  | *PLAU* | Plasminogen activator, urokinase |
|  |  | *LUM* | Lumican |
|  |  | *MMP9* | Matrix metallopeptidase 9 |
| **D O W N** | **Neuroactive ligand-receptor interaction** | *PRLHR* | Prolactin releasing hormone receptor |
|  |  | *HRH3* | Histamine receptor H3 |
|  |  | *GABRD* | Gamma-aminobutyric acid type A receptor subunit delta |
|  |  | *NTSR2* | Neurotensin receptor 2 |
|  |  | *GRIN1* | Glutamate ionotropic receptor NMDA type subunit 1 |
|  |  | *SST* | Somatostatin |

**Supplementary Table 3. The results of the enrichment analysis using common DEGs (continued)**

**Supplementary Table 4. The median cut-off value for SOCS3 gene expression in each cohort**

|  | **TCGA**  **(range)** | **CGGA325**  **(range)** | **CGGA693**  **(range)** | **GSE16011**  **(range)** |
| --- | --- | --- | --- | --- |
| **GBM** | 3190.00  (161-18503) | 36.86  (0.89-280.55) | 19.07  (0.19-303.38) | 6.91  (5.7-9.04) |
| **LGG** | 314.50  (18-33943) | 2.21  (0.29-141.710) | 1.74  (0.050-176.18) | 6.29  (5.64-8.02) |

**Supplementary Table 5. R code in this study**

if (!"BiocManager" %in% rownames(installed.packages()))

install.packages("BiocManager")

BiocManager::install("TCGAWorkflow")

library(TCGAWorkflow)

library(TCGAbiolinks)

library(DESeq2)

library(edgeR)

library(dplyr)

library(geneSA)

##Pre-Processing Data

query <- GDCquery(project = "TCGA-GBM",

data.category = "Gene expression",

data.type = "Gene expression quantification",

platform = "Illumina HiSeq",

file.type = "results",

sample.type = "Primary Tumor",

legacy = TRUE)

GDCdownload(query)

query$results

gbm.exp <- GDCprepare(query,

save = TRUE,

summarizedExperiment = TRUE,

save.filename = "GBMIllumina_HiSeq.rda")#156

# get indexed clinical patient data for GBM samples

gbm_clin <- GDCquery_clinic(project = "TCGA-GBM", type = "Clinical")

write.csv(gbm_clin,"TCGA_GBM_clinic.csv")

table(gbm.exp$vital_status)

query <- GDCquery(project = "TCGA-LGG",

data.category = "Gene expression",

data.type = "Gene expression quantification",

platform = "Illumina HiSeq",

file.type = "results",

sample.type = "Primary Tumor",

legacy = TRUE)

query$results

GDCdownload(query)

lgg.exp <- GDCprepare(query,

save = TRUE,

summarizedExperiment = TRUE,

save.filename = "LGGIllumina_HiSeq.rda")#516

# get indexed clinical patient data for LGG samples

lgg_clin <- GDCquery_clinic(project = "TCGA-LGG", type = "Clinical")

write.csv(lgg_clin,"TCGA_LGG_clinic.csv")

#load("LGGIllumina_HiSeq.rda")

#load("GBMIllumina_HiSeq.rda")

##data Preprocessing

dataPrep_LGG <- TCGAanalyze_Preprocessing(object = lgg.exp,

cor.cut = 0.6,

datatype = "raw_count",

filename = "LGG_IlluminaHiSeq_RNASeqV2.png")

head(dataPrep_LGG)[1:5,1:5]

dataPrep_GBM <- TCGAanalyze_Preprocessing(object = gbm.exp,

cor.cut = 0.6,

datatype = "raw_count",

filename = "GBM_IlluminaHiSeq_RNASeqV2.png")

#write.csv(dataPrep_LGG, "TCGA_LGG_exp.csv")

#write.csv(dataPrep_GBM,"TCGA_GBM_exp.csv")

dataNorm <- TCGAanalyze_Normalization(tabDF = cbind(dataPrep_LGG, dataPrep_GBM),

geneInfo = TCGAbiolinks::geneInfo,

method = "gcContent") #19866 672

dataFilt <- TCGAanalyze_Filtering(tabDF = dataNorm,

method = "quantile",

qnt.cut = 0.25) # 14899 672

save(dataFilt, file = paste0("LGG_GBM_Norm_IlluminaHiSeq.rda"))

colnames(dataFilt)

rownames(dataFilt)

write.csv(dataFilt,"LGG_GBM_Norm.csv")

dataFiltLGG <- subset(dataFilt, select = substr(colnames(dataFilt),1,12) %in% lgg_clin$bcr_patient_barcode)

dataFiltGBM <- subset(dataFilt, select = substr(colnames(dataFilt),1,12) %in% gbm_clin$bcr_patient_barcode)

?TCGAanalyze_DEA

# differentially expressed genes (DEG) with exact test

dataDEGs_exat <- TCGAanalyze_DEA(mat1 = dataFiltLGG,

mat2 = dataFiltGBM,

Cond1type = "LGG",

Cond2type = "GBM",

fdr.cut = 0.05 ,

logFC.cut = 1.5,

method = 'exactTest' )

?TCGAanalyze_DEA

?TCGAanalyze_DEA()

write.csv(dataDEGs_exat, "TCGA_LGG_GBM_DEGs.csv")

sig.Gene<-rownames(dataDEGs_exat)

nor.LGG<-as.data.frame(dataFiltLGG, row.names = rownames(dataFiltLGG),colnames(dataFiltLGG))

nor.GBM<-as.data.frame(dataFiltGBM, row.names = rownames(dataFiltGBM),colnames(dataFiltGBM))

sig.LGG<-nor.LGG[sig.Gene,]

sig.GBM<-nor.GBM[sig.Gene,]

colnames(sig.GBM)

sig.LGG$Gene<-rownames(sig.LGG)

sig.GBM$Gene<-rownames(sig.GBM)

LGG.GBM<-merge(sig.LGG,sig.GBM, by="Gene")

dim(LGG.GBM)

colnames(LGG.GBM)

rownames(LGG.GBM)<-LGG.GBM$Gene

LGG.GBM<-LGG.GBM[,-1]

T.LGG.GBM<-as.data.frame(t(LGG.GBM))

T.LGG.GBM$type<-c(rep("LGG",516),rep("GBM",156))

lgg_clin[1]

lgg_clin[1]

rownames(T.LGG.GBM)[1:5]

a<-rownames(T.LGG.GBM)

b<-substr(a, 1,12)

T.LGG.GBM$barcode<-b

LGG.clin<-select(lgg_clin,bcr_patient_barcode,vital_status,gender,days_to_death,days_to_last_follow_up,disease)

GBM.clin<-select(gbm_clin,bcr_patient_barcode,vital_status,gender,days_to_death,days_to_last_follow_up,disease)

clin<-rbind(LGG.clin,GBM.clin)

dim(clin)

colnames(lgg_clin)

table(gbm_clin[,121])

table(lgg_clin[,41])

lgg.gbm.cli<- clin[which(clin$bcr_patient_barcode %in% b),]

colnames(lgg.gbm.cli)[1]<-"barcode"

final_df<-merge(lgg.gbm.cli,T.LGG.GBM,by="barcode")

write.csv(final_df,"pre_survival.csv")

###############CGGA dataset

# read RNA file

CGGA.325.exp<- read.csv("CGGA.mRNAseq_325_exp.csv",header = T, stringsAsFactors = F)

CGGA.325.cli<- read.csv("CGGA.mRNAseq_325_clinical.csv",header = T, stringsAsFactors = F)

CGGA.693.exp<- read.csv("CGGA.mRNAseq_693_exp.csv",header = T, stringsAsFactors = F)

CGGA.693.cli<- read.csv("CGGA.mRNAseq_693_clinical.csv",header = T, stringsAsFactors = F)

colnames(CGGA.325.cli)[1]<-"CGGA_ID"

colnames(CGGA.693.cli)[1]<-"CGGA_ID"

Pri.cli.3<-CGGA.325.cli[which(CGGA.325.cli$PRS_type=="Primary"),]

Pri.cli.6<-CGGA.693.cli[which(CGGA.693.cli$PRS_type=="Primary"),]

table(Pri.cli.3$Gender)

rownames(Pri.cli.3)<-Pri.cli.3$CGGA_ID

rownames(Pri.cli.6)<-Pri.cli.6$CGGA_ID

colnames(CGGA.325.exp)[1]<-"Gene_Name"

colnames(CGGA.693.exp)[1]<-"Gene_Name"

rownames(CGGA.325.exp)<-CGGA.325.exp$Gene_Name

rownames(CGGA.693.exp)<-CGGA.693.exp$Gene_Name

Pri.exp.3<-subset(CGGA.325.exp, select= c("Gene_Name",Pri.cli.3$CGGA_ID))

Pri.exp.6<-subset(CGGA.693.exp, select= c("Gene_Name",Pri.cli.6$CGGA_ID))

12/276

colnames(Pri.exp.3)

colnames(Pri.exp.6)

rownames(Pri.exp.3)

cli.df.3<-data.frame(ID=Pri.cli.3$CGGA_ID, Group=ifelse(Pri.cli.3$Histology=="GBM","GBM","LGG"))

cli.df.6<-data.frame(ID=Pri.cli.6$CGGA_ID, Group=ifelse(Pri.cli.6$Histology=="GBM","GBM","LGG"))

table(cli.df.3$Group)

Pri.exp.3$Gene_Name

d_obj3 <- DGEList(counts=Pri.exp.3[,2:230], group =cli.df.3$Group )

d_obj6 <- DGEList(counts=Pri.exp.6[,2:423], group =cli.df.6$Group)

pseudoCounts3 <- log2(d_obj3$counts+1)

head(pseudoCounts3)

pseudoCounts6 <- log2(d_obj6$counts+1)

head(pseudoCounts6)

hist(pseudoCounts3[,9])

boxplot(pseudoCounts3, col="gray", las=3)

dgeFull3 <- DGEList(d_obj3$counts[apply(d_obj3$counts, 1, sum) != 0, ],

group=d_obj3$samples$group)

dgeFull6 <- DGEList(d_obj6$counts[apply(d_obj6$counts, 1, sum) != 0, ],

group=d_obj6$samples$group)

head(dgeFull3$counts)

dgeFull3 <- calcNormFactors(dgeFull3, method="TMM")

dgeFull6 <- calcNormFactors(dgeFull6, method="TMM")

eff.lib.size3 <- dgeFull3$samples$lib.size*dgeFull3$samples$norm.factors

eff.lib.size6 <- dgeFull6$samples$lib.size*dgeFull6$samples$norm.factors

normCounts3 <- cpm(dgeFull3)

normCounts6 <- cpm(dgeFull6)

pseudoNormCounts3 <- log2(normCounts3 + 1)

boxplot(pseudoNormCounts3, col="gray", las=3)

pseudoNormCounts6 <- log2(normCounts6 + 1)

boxplot(pseudoNormCounts6, col="gray", las=3)

dgeFull3 <- estimateCommonDisp(dgeFull3)

dgeFull3 <- estimateTagwiseDisp(dgeFull3)

dgeFull3

dgeFull6 <- estimateCommonDisp(dgeFull6)

dgeFull6 <- estimateTagwiseDisp(dgeFull6)

dgeFull6

dgeTest3 <- exactTest(dgeFull3)

dgeTest3

df.dgeTest.3<-dgeTest3$table

dgeTest6 <- exactTest(dgeFull6)

dgeTest6

df.dgeTest.6<-dgeTest6$table

resNoFilt3 <- topTags(dgeTest3, n=nrow(dgeTest3$table))

resNoFilt6 <- topTags(dgeTest6, n=nrow(dgeTest6$table))

resCGGA325<-resNoFilt3$table

resCGGA693<-resNoFilt6$table

##selection of significant gene

sig.CGGA325<-resCGGA325[resCGGA325$FDR<0.05 & abs(resCGGA325$logFC)>1.5,]

sig.CGGA693<-resCGGA693[resCGGA693$FDR<0.05 & abs(resCGGA693$logFC)>1.5,]

write.csv(sig.CGGA325,"res_CGGA325_result.csv")

write.csv(sig.CGGA693,"res_CGGA693_result.csv")

#####Univariate Cox regression of TCGA

TCGA <- read.csv("pre_survival.csv", row.names=1)

colnames(TCGA)

###days to death's NA chage to data to last follow up in alive sample

a<-TCGA$days_to_last_follow_up

b<-TCGA$days_to_death

c<-which(is.na(b))

b[is.na(b)]<-a[c]

TCGA$days_to_death<-b

TCGA_F<-TCGA[-c(which(TCGA$vital_status=="Not Reported")),]

dim(TCGA)

dim(TCGA_F)

colnames(TCGA_F)[1]<-"State"

##cohort split

TCGA.GBM<-TCGA_F %>% filter(disease=="GBM")

TCGA.LGG<-TCGA_F %>% filter(disease=="LGG")

## data check

colnames(TCGA)

TCGA.GBM$State<-factor(TCGA.GBM$State)

TCGA.LGG$State<-factor(TCGA.LGG$State)

###median table

TCGA.GBM.gene<-TCGA.GBM[,colnames(TCGA.GBM) %in% str_replace(DEGs$gene,"-",".")]

TCGA.LGG.gene<-TCGA.LGG[,colnames(TCGA.LGG) %in% str_replace(DEGs$gene,"-",".")]

cut.GBM <- apply(TCGA.GBM.gene,2, function(x) ifelse(x > median(x),"up","down")) %>% as.data.frame()

cut.LGG <- apply(TCGA.LGG.gene,2, function(x) ifelse(x > median(x),"up","down")) %>% as.data.frame()

#median datatable

New.df.GBM<-data.frame(State=factor(TCGA.GBM$State),Time=TCGA.GBM$days_to_death, gender=factor(TCGA.GBM$gender), cut.GBM)

New.df.LGG<-data.frame(State=factor(TCGA.LGG$State),Time=TCGA.LGG$days_to_death, gender=factor(TCGA.LGG$gender), cut.LGG)

colnames(TCGA.LGG)

##cox regression

cox.res.GBM<-data.frame(matrix(nrow=218, ncol=6))

colnames(cox.res.GBM)<-c("gene","Hazard","lowerCI","upperCI","p.val","median")

cox.res.GBM

colnames(New.df.GBM)

for (i in 4:221 ){

surv.fit <- Surv(New.df.GBM$Time, New.df.GBM$State=="Dead")

surv.fit2 = coxph(surv.fit ~ New.df.GBM[,i], na.action=na.omit, data=New.df.GBM)

a<-summary(surv.fit2)

cox.res.GBM[i-3,1]<-colnames(New.df.GBM)[i]

cox.res.GBM[i-3,5]<-a$coefficients[,5]

cox.res.GBM[i-3,2]<-a$conf.int[,1]

cox.res.GBM[i-3,3]<-a$conf.int[,3]

cox.res.GBM[i-3,4]<-a$conf.int[,4]

}

cox.res.GBM[cox.res.GBM$gene=="SOCS3",]

table(cox.res.GBM$p.val<0.05)

cox.res.GBM<-cox.res.GBM[order(cox.res.GBM$p.val,decreasing = F),]

cox.res.LGG<-data.frame(matrix(nrow=218, ncol=6))

colnames(cox.res.LGG)<-c("gene","Hazard","lowerCI","upperCI","p.val","median")

for (i in 4:221 ){

surv.fit <- Surv(New.df.LGG$Time, New.df.LGG$State=="Dead")

surv.fit2 = coxph(surv.fit ~ New.df.LGG[,i], na.action=na.omit, data=New.df.LGG)

a<-summary(surv.fit2)

cox.res.LGG[i-3,1]<-colnames(New.df.LGG)[i]

cox.res.LGG[i-3,5]<-a$coefficients[,5]

cox.res.LGG[i-3,2]<-a$conf.int[,1]

cox.res.LGG[i-3,3]<-a$conf.int[,3]

cox.res.LGG[i-3,4]<-a$conf.int[,4]

}

cox.res.LGG

table(cox.res.LGG$p.val<0.05)

cox.res.LGG<-cox.res.LGG[order(cox.res.LGG$p.val,decreasing = F),]

cox.res.LGG[cox.res.LGG$gene=="SOCS3",]

intersect(cox.res.LGG[cox.res.LGG$p.val<0.05,]$gene,cox.res.GBM[cox.res.GBM$p.val<0.05,]$gene)

#######Univariate Cox regression of CGGA

getwd()

DEGs<-read.csv("CommonDEGs.csv",header = T, stringsAsFactors = F)

upGenes<-DEGs %>% filter(DEG=="up") %>% select(gene)

downGenes<-DEGs %>% filter(DEG=="down") %>% select(gene)

t.exp.3<-t(Pri.exp.3[DEGs$gene,-1])

colnames(Pri.exp.3)

LGG3<-cli.df.3[cli.df.3$Group=="LGG",1]

OS.MONTHS.LGG3<-Pri.cli.3[Pri.cli.3$CGGA_ID %in% LGG3,c(1,5,6,7,8,11,12)]

GBM3<-cli.df.3[cli.df.3$Group=="GBM",1]

OS.MONTHS.GBM3<-Pri.cli.3[Pri.cli.3$CGGA_ID %in% GBM3,c(1,7,6,7,8,11,12)]

t.exp.6<-t(Pri.exp.6[DEGs$gene,-1])

colnames(Pri.exp.6)

LGG6<-cli.df.6[cli.df.6$Group=="LGG",1]

OS.MONTHS.LGG6<-Pri.cli.6[Pri.cli.6$CGGA_ID %in% LGG6,c(1,5,6,7,8,11,12)]

GBM6<-cli.df.6[cli.df.6$Group=="GBM",1]

OS.MONTHS.GBM6<-Pri.cli.6[Pri.cli.6$CGGA_ID %in% GBM6,c(1,5,6,7,8,11,12)]

all(rownames(t.exp.6)==OS.MONTHS.LGG6)

exp.LGG.3<-t.exp.3[OS.MONTHS.LGG3$CGGA_ID,]

exp.GBM.3<-t.exp.3[OS.MONTHS.GBM3$CGGA_ID,]

exp.LGG.6<-t.exp.6[OS.MONTHS.LGG6$CGGA_ID,]

exp.GBM.6<-t.exp.6[OS.MONTHS.GBM6$CGGA_ID,]

exp.LGG.3.1 <- apply(exp.LGG.3,2, function(x) ifelse(x > median(x),"up","down")) %>% as.data.frame()

exp.GBM.3.1 <- apply(exp.GBM.3,2, function(x) ifelse(x > median(x),"up","down")) %>% as.data.frame()

exp.LGG.6.1 <- apply(exp.LGG.6,2, function(x) ifelse(x > median(x),"up","down")) %>% as.data.frame()

exp.GBM.6.1 <- apply(exp.GBM.6,2, function(x) ifelse(x > median(x),"up","down")) %>% as.data.frame()

rownames(exp.LGG.3.1)[1:10]

OS.MONTHS.LGG3$CGGA_ID[1:10]

New.df.C3.GBM<-data.frame(State=factor(OS.MONTHS.GBM3$Censor),Time=OS.MONTHS.GBM3$OS, gender=factor(OS.MONTHS.GBM3$Age), exp.GBM.3.1)

New.df.C3.LGG<-data.frame(State=factor(OS.MONTHS.LGG3$Censor),Time=OS.MONTHS.LGG3$OS, gender=factor(OS.MONTHS.LGG3$Age), exp.LGG.3.1)

New.df.C6.GBM<-data.frame(State=factor(OS.MONTHS.GBM6$Censor),Time=OS.MONTHS.GBM6$OS, gender=factor(OS.MONTHS.GBM6$Age), exp.GBM.6.1)

New.df.C6.LGG<-data.frame(State=factor(OS.MONTHS.LGG6$Censor),Time=OS.MONTHS.LGG6$OS, gender=factor(OS.MONTHS.LGG6$Age), exp.LGG.6.1)

table(is.na(New.df.C3.GBM$State))

cox.res.C3.GBM<-data.frame(matrix(nrow=218, ncol=6))

colnames(cox.res.C3.GBM)<-c("gene","Hazard","lowerCI","upperCI","p.val","median")

colnames(New.df.C3.GBM)

dim(New.df.C3.GBM)

for (i in 4:221 ){

surv.fit <- Surv(New.df.C3.GBM$Time, New.df.C3.GBM$State==1)

surv.fit2 = coxph(surv.fit ~ New.df.C3.GBM[,i], na.action=na.omit, data=New.df.C3.GBM)

a<-summary(surv.fit2)

cox.res.C3.GBM[i-3,1]<-colnames(New.df.C3.GBM)[i]

cox.res.C3.GBM[i-3,5]<-a$coefficients[,5]

cox.res.C3.GBM[i-3,2]<-a$conf.int[,1]

cox.res.C3.GBM[i-3,3]<-a$conf.int[,3]

cox.res.C3.GBM[i-3,4]<-a$conf.int[,4]

}

cox.res.C3.GBM[cox.res.C3.GBM$gene=="SOCS3",]

cox.res.C3.LGG<-data.frame(matrix(nrow=218, ncol=6))

colnames(cox.res.C3.LGG)<-c("gene","Hazard","lowerCI","upperCI","p.val","median")

colnames(New.df.C3.LGG)

dim(New.df.C3.LGG)

for (i in 4:221 ){

surv.fit <- Surv(New.df.C3.LGG$Time, New.df.C3.LGG$State==1)

surv.fit2 = coxph(surv.fit ~ New.df.C3.LGG[,i], na.action=na.omit, data=New.df.C3.LGG)

a<-summary(surv.fit2)

cox.res.C3.LGG[i-3,1]<-colnames(New.df.C3.LGG)[i]

cox.res.C3.LGG[i-3,5]<-a$coefficients[,5]

cox.res.C3.LGG[i-3,2]<-a$conf.int[,1]

cox.res.C3.LGG[i-3,3]<-a$conf.int[,3]

cox.res.C3.LGG[i-3,4]<-a$conf.int[,4]

}

cox.res.C3.LGG[cox.res.C3.LGG$gene=="SOCS3",]

cox.res.C6.GBM<-data.frame(matrix(nrow=218, ncol=6))

colnames(cox.res.C6.GBM)<-c("gene","Hazard","lowerCI","upperCI","p.val","median")

colnames(New.df.C6.GBM)

dim(New.df.C6.GBM)

for (i in 4:221 ){

surv.fit <- Surv(New.df.C6.GBM$Time, New.df.C6.GBM$State==1)

surv.fit2 = coxph(surv.fit ~ New.df.C6.GBM[,i], na.action=na.omit, data=New.df.C6.GBM)

a<-summary(surv.fit2)

cox.res.C6.GBM[i-3,1]<-colnames(New.df.C6.GBM)[i]

cox.res.C6.GBM[i-3,5]<-a$coefficients[,5]

cox.res.C6.GBM[i-3,2]<-a$conf.int[,1]

cox.res.C6.GBM[i-3,3]<-a$conf.int[,3]

cox.res.C6.GBM[i-3,4]<-a$conf.int[,4]

}

cox.res.C6.GBM[cox.res.C6.GBM$gene=="SOCS3",]

cox.res.C6.LGG<-data.frame(matrix(nrow=218, ncol=6))

colnames(cox.res.C6.LGG)<-c("gene","Hazard","lowerCI","upperCI","p.val","median")

colnames(New.df.C6.LGG)

dim(New.df.C6.LGG)

time=New.df.C6.LGG$Time

status=New.df.C6.LGG$State

for (i in 4:221 ){

surv.fit <- Surv(New.df.C6.LGG$Time, New.df.C6.LGG$State==1)

surv.fit2 = coxph(surv.fit ~ New.df.C6.LGG[,i], na.action=na.omit, data=New.df.C6.LGG)

a<-summary(surv.fit2)

cox.res.C6.LGG[i-3,1]<-colnames(New.df.C6.LGG)[i]

cox.res.C6.LGG[i-3,5]<-a$coefficients[,5]

cox.res.C6.LGG[i-3,2]<-a$conf.int[,1]

cox.res.C6.LGG[i-3,3]<-a$conf.int[,3]

cox.res.C6.LGG[i-3,4]<-a$conf.int[,4]

}

cox.res.C6.LGG[cox.res.C6.LGG$gene=="SOCS3",]

surv_diff <- survdiff(Surv(time, status) ~ sex, data = lung)

surv_diff

####cox regression with clinical

T.GBM.cli<-read.csv("TCGA_GBM_clinic.csv",header = T, stringsAsFactors = F)

T.LGG.cli<-read.csv("TCGA_LGG_clinic.csv",header = T, stringsAsFactors = F)

colnames(T.GBM.cli)

head(T.GBM.cli)

a<-T.GBM.cli$days_to_last_follow_up

b<-T.GBM.cli$days_to_death

c<-which(is.na(b))

b[is.na(b)]<-a[c]

T.GBM.cli$days_to_death<-b

T.GBM.cli[c,]

T.GBM.cli_F<-T.GBM.cli[-c(which(T.GBM.cli$vital_status=="Not Reported"))]

table(T.GBM.cli_F$treatments_pharmaceutical_treatment_or_therapy)

a<-T.LGG.cli$days_to_last_follow_up

b<-T.LGG.cli$days_to_death

c<-which(is.na(b))

b[is.na(b)]<-a[c]

T.LGG.cli$days_to_death<-b

T.LGG.cli_F<-T.LGG.cli[-c(which(T.LGG.cli$vital_status=="Not Reported"))]

dim(TCGA)

# read RNA file

C.325.cli<- read.csv("CGGA.mRNAseq_325_clinical.csv",header = T, stringsAsFactors = F)

C.693.cli<- read.csv("CGGA.mRNAseq_693_clinical.csv",header = T, stringsAsFactors = F)

colnames(C.325.cli)[1]<-"CGGA_ID"

colnames(C.693.cli)[1]<-"CGGA_ID"

table(C.325.cli$Histology)

table(C.325.cli$PRS_type)

C.3.GBM<-C.325.cli %>% filter(PRS_type=="Primary") %>% filter(Histology=="GBM")

C.3.LGG<-C.325.cli %>% filter(PRS_type=="Primary") %>% filter(!str_detect(Histology,"GBM"))

C.6.GBM<-C.693.cli %>% filter(PRS_type=="Primary") %>% filter(Histology=="GBM")

C.6.LGG<-C.693.cli %>% filter(PRS_type=="Primary") %>% filter(!str_detect(Histology,"GBM"))

colnames(C.3.GBM)

fit <- survfit(Surv(OS, Censor) ~ Gender, data = C.3.GBM)

colnames(C.3.GBM)

#CGGA3_GBM

surv.fit <- Surv(C.3.GBM$OS, C.3.GBM$Censor==1)

surv.gender.C3.GBM = coxph(surv.fit ~ C.3.GBM$Gender, na.action=na.omit, data=C.3.GBM)

summary(surv.gender.C3.GBM)

surv.age.C3.GBM = coxph(surv.fit ~ C.3.GBM$Age, na.action=na.omit, data=C.3.GBM)

summary(surv.age.C3.GBM) #0.044

surv.Radio.C3.GBM = coxph(surv.fit ~ C.3.GBM$Radio_status, na.action=na.omit, data=C.3.GBM)

summary(surv.Radio.C3.GBM)

surv.Chemo.C3.GBM = coxph(surv.fit ~ C.3.GBM$Chemo_status, na.action=na.omit, data=C.3.GBM)

summary(surv.Chemo.C3.GBM)#p-value<0.001

#CGGA6_GBM

surv.fit <- Surv(C.6.GBM$OS, C.6.GBM$Censor==1)

surv.gender.C6.GBM = coxph(surv.fit ~ C.6.GBM$Gender, na.action=na.omit, data=C.6.GBM)

summary(surv.gender.C6.GBM)

surv.age.C6.GBM = coxph(surv.fit ~ C.6.GBM$Age, na.action=na.omit, data=C.6.GBM)

summary(surv.age.C6.GBM) #p-value 0.008

surv.Radio.C6.GBM = coxph(surv.fit ~ C.6.GBM$Radio_status, na.action=na.omit, data=C.6.GBM)

summary(surv.Radio.C6.GBM)#p-value <0.001

surv.Chemo.C6.GBM = coxph(surv.fit ~ C.6.GBM$Chemo_status, na.action=na.omit, data=C.6.GBM)

summary(surv.Chemo.C6.GBM)#p-value 0.003

#CGGA3_LGG

surv.fit <- Surv(C.3.LGG$OS, C.3.LGG$Censor==1)

surv.gender.C3.LGG = coxph(surv.fit ~ C.3.LGG$Gender, na.action=na.omit, data=C.3.LGG)

summary(surv.gender.C3.LGG)

surv.age.C3.LGG = coxph(surv.fit ~ C.3.LGG$Age, na.action=na.omit, data=C.3.LGG)

summary(surv.age.C3.LGG) #p-value<0.001

surv.Radio.C3.LGG = coxph(surv.fit ~ C.3.LGG$Radio_status, na.action=na.omit, data=C.3.LGG)

summary(surv.Radio.C3.LGG)

surv.Chemo.C3.LGG = coxph(surv.fit ~ C.3.LGG$Chemo_status, na.action=na.omit, data=C.3.LGG)

summary(surv.Chemo.C3.LGG)#p-value<0.001

#CGGA6_LGG

surv.fit <- Surv(C.6.LGG$OS, C.6.LGG$Censor==1)

surv.gender.C6.LGG = coxph(surv.fit ~ C.6.LGG$Gender, na.action=na.omit, data=C.6.LGG)

summary(surv.gender.C6.LGG)

surv.age.C6.LGG = coxph(surv.fit ~ C.6.LGG$Age, na.action=na.omit, data=C.6.LGG)

summary(surv.age.C6.LGG)

surv.Radio.C6.LGG = coxph(surv.fit ~ C.6.LGG$Radio_status, na.action=na.omit, data=C.6.LGG)

summary(surv.Radio.C6.LGG)

surv.Chemo.C6.LGG = coxph(surv.fit ~ C.6.LGG$Chemo_status, na.action=na.omit, data=C.6.LGG)

summary(surv.Chemo.C6.LGG)

#TCGA_GBM

surv.fit <- Surv(T.GBM.cli_F$days_to_death, T.GBM.cli_F$vital_status=="Dead")

surv.gender.TCGA.GBM = coxph(surv.fit ~ T.GBM.cli_F$gender, na.action=na.omit, data=T.GBM.cli_F)

summary(surv.gender.TCGA.GBM)#p-value 0.04

surv.age.TCGA.GBM = coxph(surv.fit ~ T.GBM.cli_F$age_at_index, na.action=na.omit, data=T.GBM.cli_F)

summary(surv.age.TCGA.GBM) #p-value 0.001

surv.Radio.TCGA.GBM = coxph(surv.fit ~ T.GBM.cli_F$treatments_radiation_treatment_or_therapy, na.action=na.omit, data=T.GBM.cli_F)

summary(surv.Radio.TCGA.GBM)#p-value <0.001

surv.Chemo.TCGA.GBM = coxph(surv.fit ~ T.GBM.cli_F$treatments_pharmaceutical_treatment_or_therapy, na.action=na.omit, data=T.GBM.cli_F)

summary(surv.Chemo.TCGA.GBM)#p-value 0.003

#CGGA3_LGG

surv.fit <- Surv(C.3.LGG$OS, C.3.LGG$Censor==1)

surv.gender.C3.LGG = coxph(surv.fit ~ C.3.LGG$Gender, na.action=na.omit, data=C.3.LGG)

summary(surv.gender.C3.LGG)

surv.age.C3.LGG = coxph(surv.fit ~ C.3.LGG$Age, na.action=na.omit, data=C.3.LGG)

summary(surv.age.C3.LGG) #p-value<0.001

surv.Radio.C3.LGG = coxph(surv.fit ~ C.3.LGG$Radio_status, na.action=na.omit, data=C.3.LGG)

summary(surv.Radio.C3.LGG)

surv.Chemo.C3.LGG = coxph(surv.fit ~ C.3.LGG$Chemo_status, na.action=na.omit, data=C.3.LGG)

summary(surv.Chemo.C3.LGG)#p-value<0.001

######multivariate cox regression

surv.fit<-Surv(TCGA.LGG.1$survival,TCGA.LGG.1$status)

surv.fit2<-coxph(surv.fit~TCGA.LGG.1$cutoff,na.action = na.omit, data=TCGA.LGG.1)

res.LGG.SOCS3<-summary(surv.fit2)

surv.fit2<-coxph(surv.fit~TCGA.LGG.1$IDH1_status,na.action = na.omit, data=TCGA.LGG.1)

res.LGG.IDH1<-summary(surv.fit2)

surv.fit2<-coxph(surv.fit~TCGA.LGG.1$IDH1_status+TCGA.LGG.1$cutoff+TCGA.LGG.1$co_del_1p_19q,na.action = na.omit, data=TCGA.LGG.1)

surv.fit2<-coxph(surv.fit~TCGA.LGG.1$co_del_1p_19q,na.action = na.omit, data=TCGA.LGG.1)

table(TCGA.LGG.1$status,TCGA.LGG.1$co_del_1p_19q)

summary(surv.fit2)

colnames(TCGA.LGG.1)[16]

surv.fit<-Surv(TCGA.GBM.1$survival,TCGA.GBM.1$status)

surv.fit2<-coxph(surv.fit~TCGA.GBM.1$cutoff,na.action = na.omit, data=TCGA.GBM.1)

res.GBM.SOCS3<-summary(surv.fit2)

surv.fit2<-coxph(surv.fit~TCGA.GBM.1$IDH1_status,na.action = na.omit, data=TCGA.GBM.1)

res.GBM.IDH1<-summary(surv.fit2)

surv.fit2<-coxph(surv.fit~TCGA.GBM.1$IDH1_status+TCGA.GBM.1$cutoff,na.action = na.omit, data=TCGA.GBM.1)

res.GBM.multi<-summary(surv.fit2)

TCGA.LGG.1$IDH1_status

CGGA_32<-read.csv("preSurviveCGGA32.csv",header = T, stringsAsFactors = F)

CGGA_69<-read.csv("preSurviveCGGA69.csv",header = T, stringsAsFactors = F)

colnames(CGGA_32)

Pr.CGGA32<-CGGA_32 %>% filter(PRS_type=="Primary")

Pr.CGGA69<-CGGA_69 %>% filter(PRS_type=="Primary")

Pr.CGGA32$Histology2<-ifelse(Pr.CGGA32$Histology=="GBM","GBM","LGG")

Pr.CGGA69$Histology2<-ifelse(Pr.CGGA69$Histology=="GBM","GBM","LGG")

colnames(Pr.CGGA32)[1:20]

CGGA.32.1<-Pr.CGGA32[,c("X","Histology2","SOCS3","IDH_mutation_status","X1p19q_codeletion_status","OS","Censor" )]

table(CGGA.32.1$X1p19q_codeletion_status)

CGGA.32.1$codel<-ifelse(CGGA.32.1$X1p19q_codeletion_status=="Codel","TRUE","FALSE")

CGGA.69.1<-Pr.CGGA69[,c("X","Histology2","SOCS3","IDH_mutation_status","X1p19q_codeletion_status","OS","Censor" )]

CGGA.69.1$codel<-ifelse(CGGA.69.1$X1p19q_codeletion_status=="Codel","TRUE","FALSE")

CGGA.32.LGG<-CGGA.32.1 %>% filter(Histology2=="LGG")

CGGA.32.GBM<-CGGA.32.1 %>% filter(Histology2=="GBM")

CGGA.69.LGG<-CGGA.69.1 %>% filter(Histology2=="LGG")

CGGA.69.GBM<-CGGA.69.1 %>% filter(Histology2=="GBM")

CGGA.32.LGG$cutoff<-factor(ifelse(median(CGGA.32.LGG$SOCS3)>=CGGA.32.LGG$SOCS3,"Low","High"),levels = c("Low","High"))

CGGA.69.LGG$cutoff<-factor(ifelse(median(CGGA.69.LGG$SOCS3)>=CGGA.69.LGG$SOCS3,"Low","High"),levels = c("Low","High"))

CGGA.32.GBM$cutoff<-factor(ifelse(median(CGGA.32.GBM$SOCS3)>=CGGA.32.GBM$SOCS3,"Low","High"),levels = c("Low","High"))

CGGA.69.GBM$cutoff<-factor(ifelse(median(CGGA.69.GBM$SOCS3)>=CGGA.69.GBM$SOCS3,"Low","High"),levels = c("Low","High"))

surv.fit<-Surv(CGGA.32.LGG$OS,CGGA.32.LGG$Censor)

surv.fit2<-coxph(surv.fit~CGGA.32.LGG$cutoff,na.action = na.omit, data=CGGA.32.LGG)

res.C3.L.S<-summary(surv.fit2)

surv.fit2<-coxph(surv.fit~CGGA.32.LGG$IDH_mutation_status,na.action = na.omit, data=CGGA.32.LGG)

res.C3.L.IDH<-summary(surv.fit2)

surv.fit2<-coxph(surv.fit~CGGA.32.LGG$codel,na.action = na.omit, data=CGGA.32.LGG)

res.C3.L.codel<-summary(surv.fit2)

surv.fit2<-coxph(surv.fit~CGGA.32.LGG$IDH_mutation_status+CGGA.32.LGG$cutoff+CGGA.32.LGG$codel,na.action = na.omit, data=CGGA.32.LGG)

res.C3.L.multi<-summary(surv.fit2)

surv.fit<-Surv(CGGA.32.GBM$OS,CGGA.32.GBM$Censor)

surv.fit2<-coxph(surv.fit~CGGA.32.GBM$cutoff,na.action = na.omit, data=CGGA.32.GBM)

res.C3.G.S<-summary(surv.fit2)

surv.fit2<-coxph(surv.fit~CGGA.32.GBM$IDH_mutation_status,na.action = na.omit, data=CGGA.32.GBM)

res.C3.G.IDH<-summary(surv.fit2)

surv.fit2<-coxph(surv.fit~CGGA.32.GBM$codel,na.action = na.omit, data=CGGA.32.GBM)

res.C3.G.IDH<-summary(surv.fit2)

surv.fit2<-coxph(surv.fit~CGGA.32.GBM$IDH_mutation_status+CGGA.32.GBM$cutoff,na.action = na.omit, data=CGGA.32.GBM)

res.C3.G.multi<-summary(surv.fit2)

surv.fit<-Surv(CGGA.69.LGG$OS,CGGA.69.LGG$Censor)

surv.fit2<-coxph(surv.fit~CGGA.69.LGG$cutoff,na.action = na.omit, data=CGGA.69.LGG)

res.C6.L.S<-summary(surv.fit2)

surv.fit2<-coxph(surv.fit~CGGA.69.LGG$IDH_mutation_status,na.action = na.omit, data=CGGA.69.LGG)

res.C6.L.IDH<-summary(surv.fit2)

surv.fit2<-coxph(surv.fit~CGGA.69.LGG$codel,na.action = na.omit, data=CGGA.32.LGG)

res.C6.L.codel<-summary(surv.fit2)

table(CGGA.32.LGG$Censor,CGGA.32.LGG$codel)

surv.fit2<-coxph(surv.fit~CGGA.69.LGG$IDH_mutation_status+CGGA.69.LGG$cutoff+CGGA.69.LGG$codel,na.action = na.omit, data=CGGA.69.LGG)

res.C6.L.multi<-summary(surv.fit2)

surv.fit<-Surv(CGGA.69.GBM$OS,CGGA.69.GBM$Censor)

surv.fit2<-coxph(surv.fit~CGGA.69.GBM$cutoff,na.action = na.omit, data=CGGA.69.GBM)

res.C6.G.S<-summary(surv.fit2)

surv.fit2<-coxph(surv.fit~CGGA.69.GBM$IDH_mutation_status,na.action = na.omit, data=CGGA.69.GBM)

res.C6.G.IDH<-summary(surv.fit2)

surv.fit2<-coxph(surv.fit~CGGA.69.GBM$IDH_mutation_status+CGGA.69.GBM$cutoff,na.action = na.omit, data=CGGA.69.GBM)

res.C6.G.multi<-summary(surv.fit2)

#######GSE16011 SOCS3 validation

load("feature.GSE16011.RData")

colnames(feature1)

feature1$Description

load("pheno.GSE16011.RData")

survival<-read.csv("metaGSE16011.csv",header = T, stringsAsFactors = F)

colnames(pheno1)

title<-pheno1$title

pheno1$sampleNo<-sapply(strsplit(as.character(title)," "),"[",2)

colnames(survival)[1]<-"sampleNo"

metadata<-merge(pheno1,survival,by="sampleNo")

write.csv(metadata,"pheno16011.csv")

pheno<-read.csv("pheno16011.csv",header = T, stringsAsFactors = F)

load("expression.GSE16011.RData")

hist(gene.expression1)

colnames(gene.expression1)

rownames(gene.expression1)

colnames(pheno)

PatientSample<-pheno[!(pheno$histology.ch1=="control"),]

table(pheno$histology.ch1)

table(PatientSample$histology.ch1)

PatientSample$histology<-factor(ifelse(PatientSample$histology.ch1=="GBM (grade IV)","GBM","LGG"),levels = c("LGG","GBM"))

assession<-PatientSample$geo_accession

expression<-gene.expression1 [,assession]

expression1<-log2(expression)

intersect(colnames(expression),assession)

grpH<-PatientSample$histology

table(grpH)

design1 <- model.matrix(~0 + grpH)

rownames(design1)<-PatientSample$geo_accession

limma.expr.H <- voom(expression1, design1)$E

colnames(expression1)[1:10]

rownames(design1)[1:10]

colnames(design1) <- c("LGG","GBM")

fit1<-lmFit(expression1,design1)

cont1 <- makeContrasts(LGG-GBM,levels=design1)

fit.cont1 <- contrasts.fit(fit1,cont1)

fit.cont1 <- eBayes(fit.cont1)

res1 <- topTable(fit.cont1,number=Inf);dim(res1)

a<-res1 %>%filter(abs(logFC)>=1.0)

dim(res1 %>% filter(P.Value<0.05))

expression[rownames(expression)=="9021_at",]

rownames(a)

table(grpH)

colnames(feature1)

write.csv(feature1,"featureGSE16011.csv")

SOCS3<-"9021_at"

df<-data.frame(expression=expression[SOCS3,],grade=grpH)

df %>% filter(grade=="LGG") %>% summarise(min=min(expression),

mean=mean(expression),

median=median(expression),

max=max(expression))

df %>% filter(grade=="GBM") %>% summarise(min=min(expression),

mean=mean(expression),

median=median(expression),

max=max(expression))

#######survival analysis with geneSA

colnames(exp.LGG.3.1)<-str_replace(colnames(exp.LGG.3.1),"-",".")

colnames(exp.GBM.3.1)<-str_replace(colnames(exp.GBM.3.1),"-",".")

colnames(exp.LGG.6.1)<-str_replace(colnames(exp.LGG.6.1),"-",".")

colnames(exp.GBM.6.1)<-str_replace(colnames(exp.GBM.6.1),"-",".")

geneSA(data=exp.LGG.3.1,time = OS.MONTHS.LGG3$OS,status = OS.MONTHS.LGG3$Censor)

geneSA(data=exp.GBM.3.1,time = OS.MONTHS.GBM3$OS,status = OS.MONTHS.GBM3$Censor)

geneSA(data=exp.LGG.6.1,time = OS.MONTHS.LGG6$OS,status = OS.MONTHS.LGG6$Censor)

geneSA(data=exp.GBM.6.1,time = OS.MONTHS.GBM6$OS,status = OS.MONTHS.GBM6$Censor)

**Supplementary Materials**

**Supplementary Figure 1. Workflow for selecting differentially expressed genes (DEGs) from the RNA-seq datasets TCGA and CGGA.**

**Supplementary Figure 2.** (A) Alignment of amino acid sequences between human *SOCS3* (‘Query’) and zebrafish *socs3a* (‘Sbjct’). (B) Alignment of amino acid sequences between human *SOCS3* (‘Query’) and zebrafish *socs3b* (‘Sbjct’)

**Supplementary** **Table 1. Patient characteristics on included studies**

**Supplementary Table 2. Differentially expressed genes in LGG and GBM in all cohorts**

**Supplementary Table 3. The results of the enrichment analysis using common DEGs**

**Supplementary Table 4. The median cut-off value for SOCS3 gene expression in each cohort**

**Supplementary Table 5. R code in this study**
